# eIF4F complex dynamics are important for the activation of the integrated stress response

**DOI:** 10.1016/j.molcel.2024.04.016

**Published:** 2024-06-06

**Authors:** Kyusik Q. Kim, Ankanahalli N. Nanjaraj Urs, Victor Lasehinde, Alison C. Greenlaw, Benjamin H. Hudson, Hani S. Zaher

**Affiliations:** 1 Department of Biology, Washington University in St. Louis, St. Louis, MO 63130, USA; 2 These authors contributed equally; 3 Lead contact

## Abstract

In response to stress, eukaryotes activate the integrated stress response (ISR) via phosphorylation of eIF2α to promote the translation of pro-survival effector genes, such as *GCN4* in yeast. Complementing the ISR is the target of rapamycin (TOR) pathway, which regulates eIF4E function. Here, we probe translational control in the absence of eIF4E in *Saccharomyces cerevisiae*. Intriguingly, we find that loss of eIF4E leads to derepression of *GCN4* translation. In addition, we find that de-repression of *GCN4* translation is accompanied by neither eIF2α phosphorylation nor reduction in initiator ternary complex (TC). Our data suggest that when eIF4E levels are depleted, *GCN4* translation is de-repressed via a unique mechanism that may involve faster scanning by the small ribosome subunit due to increased local concentration of eIF4A. Overall, our findings suggest that relative levels of eIF4F components are key to ribosome dynamics and may play important roles in translational control of gene expression.

## In brief

Classically, the ISR is activated through the phosphorylation of eIF2α. Here, Kim et al. show that when eIF4E levels are depleted, the pathway is induced via a novel mechanism in which the altered ratio of heterotrimeric eIF4F complex components appears to change scanning dynamics, resulting in de-repression of *GCN4* translation.

## Graphical abstract

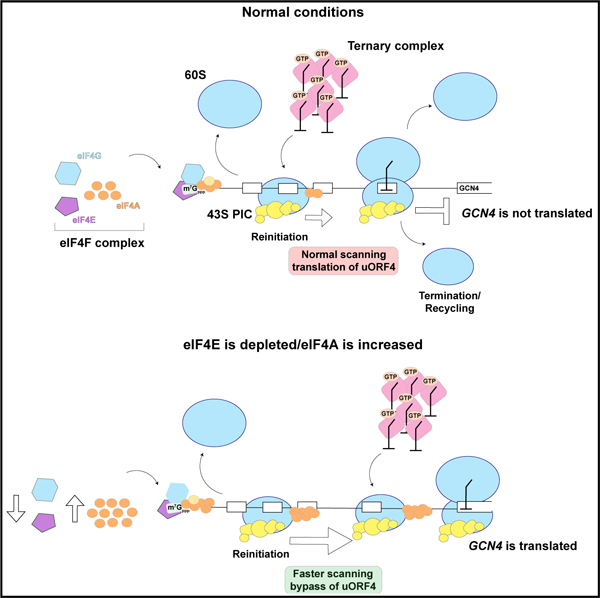

## INTRODUCTION

Careful control of which genes are expressed enables organisms to maintain homeostasis under dynamic environmental conditions. When resources are abundant, cells devote much of their energy and resources to protein synthesis.^1^ As such, translation is highly regulated to ensure protein output is properly tuned.^2^ Of the four phases of translation (initiation, elongation, termination, and recycling), initiation appears to be the most complex and regulated step.^3^ In eukaryotes, canonical initiation begins with the formation of 43S preinitiation complexes (43S PICs) by the binding of ternary complex (TC), composed of initiator methionyl-tRNA and the GTP-bound form of the factor eIF2, as well as several other initiation factors to the 40S ribosomal subunit.^4,5^ 43S PICs are then recruited to the 5′m^7^Gppp cap structure of the transcript by the eIF4F complex, composed of the cap-binding factor eIF4E, helicase eIF4A, and the scaffolding factor eIF4G. Once loaded onto the transcript, the now 48S initiation complex begins scanning for a start codon in an appropriate context.^6–10^ Upon recognition of the start codon and establishment of codon-anticodon base pairing in the P site of the 40S ribosomal subunit, the guanosine diphosphate (GDP)-bound eIF2, together with inorganic phosphate, is released.^11^ This in turn enables eIF5B to catalyze joining of the 60S ribosomal subunit to form an 80S initiating ribosome ready to engage in elongation.^12^

Although eukaryotes have evolved various mechanisms to regulate assembly and recruitment of the 80S subunit, a key conserved mechanism is the phosphorylation of Ser51 in the a subunit of eIF2 in response to various stress conditions.^13^ Phosphorylation of eIF2α activates the integrated stress response (ISR), a genetic program of survival genes that enables cells to respond to and recover from the stress.^14,15^ The phosphorylation of eIF2α is triggered by upstream kinases that monitor distinct stresses. Mammals have four such kinases: GCN2, PERK, HRI, and PKR, which are activated in response to nutrient deprivation, ER stress, cytoplasmic protein misfolding, and viral infection, respectively, while budding yeast has only one, Gcn2.^16–18^ Upon phosphorylation, eIF2 becomes a competitive inhibitor of its own guanine exchange factor (GEF) eIF2B,^19,20^ preventing the exchange of GDP for GTP, binding of initiator methionyl tRNA, and its participation in a new round of initiation. Interestingly, reduced TC levels drive increased translation of *GCN4* in yeast (ATF4 in mammals), the key effector of the ISR.^13^ Translation of *GCN4* and ATF4 are regulated through a mechanism where, under conditions of abundant TC levels, inhibitory upstream open reading frames (uORFs) in the transcript repress translation of the main ORF. When TC levels are depleted, ribosomes can bypass these inhibitory uORFs and initiate on the main ORF.^14,15^ De-repression of *GCN4* or ATF4 translation then leads to upregulation of amino acid biosynthesis and other stress-response genes.

In addition to GCN2, eukaryotes evolved an independent pathway to regulate translation in response to nutrient availability. The target of rapamycin (TOR) signaling pathway promotes growth and proliferation in response to nutrients, particularly amino acids.^21–26^ Both yeast and mammals contain two distinct complexes, TORC1 and TORC2. Of the two complexes, only TORC1 is regulated by amino acid levels and sensitive to rapamycin.^27^ In the presence of amino acids, a conserved family of RAG small GTPases form active heterodimers, bind activators of TORC1, and switch on the pathway.^21,28,29^ TOR then phosphorylates several substrates that regulate pathways such as autophagy, cytoskeleton organization, lipid metabolism, cell migration, and cell division.^30^ In addition to these pathways, TOR regulates protein synthesis at the initiation and elongation phases by directly or indirectly phosphorylating ribosomal proteins, translation factors, and translational regulators. One such target is eIF4E binding proteins (eIF4E-BPs), which sequester eIF4E away from eIF4G during nutrient deprivation and stress.^31,32^ Conversely, under nutrient-rich conditions, TOR signaling inactivates eIF4E-BPs, allowing canonical initiation to proceed.

In this study, we utilize a temperature-sensitive allele of eIF4E (*cdc33-ts4-2*)^33^ to characterize translational control in the absence of cap recognition in *Saccharomyces cerevisiae*. Using ribosome profiling, we find that loss of eIF4E leads to translation of *GCN4*. In agreement with these observations, transcriptomic and subsequent quantitative reverse transcription PCR (RT-qPCR) analyses showed induction of the Gcn4 regulon for the *cdc33-ts4-2* strain, but not the wild-type strain, at 37°C. Interestingly, immunoblot analysis of the mutant strain confirmed the accumulation of Gcn4 when eIF4E levels were depleted, but without phosphorylation of eIF2α. Our data suggest that initiation TC concentration plays a minimal role in the translation of *GCN4* when eIF4E levels are depleted. Instead, changes in relative local concentrations of eIF4A appear to be responsible for the translational control of *GCN4*. Deletion of *TIF1*, one of the two paralogs that encode eIF4A, suppresses de-repression of *GCN4* translation under both eIF4E-depleted and amino acid starvation conditions. We argue that increased local concentration of eIF4A levels enables the ribosome to bypass the inhibitory uORFs in the 5′ leader sequence of *GCN4*. Together, our findings provide a role for the relative levels of eIF4F components in translational control in general—and that of *GCN4* in particular. These observations, in turn, provide insights into a previously unappreciated mechanism for crosstalk between TOR signaling and the ISR in nutrient sensing.

## RESULTS

### Translation is greatly diminished in *cdc33-ts4-2* cells under restrictive conditions

**Figure 1.**
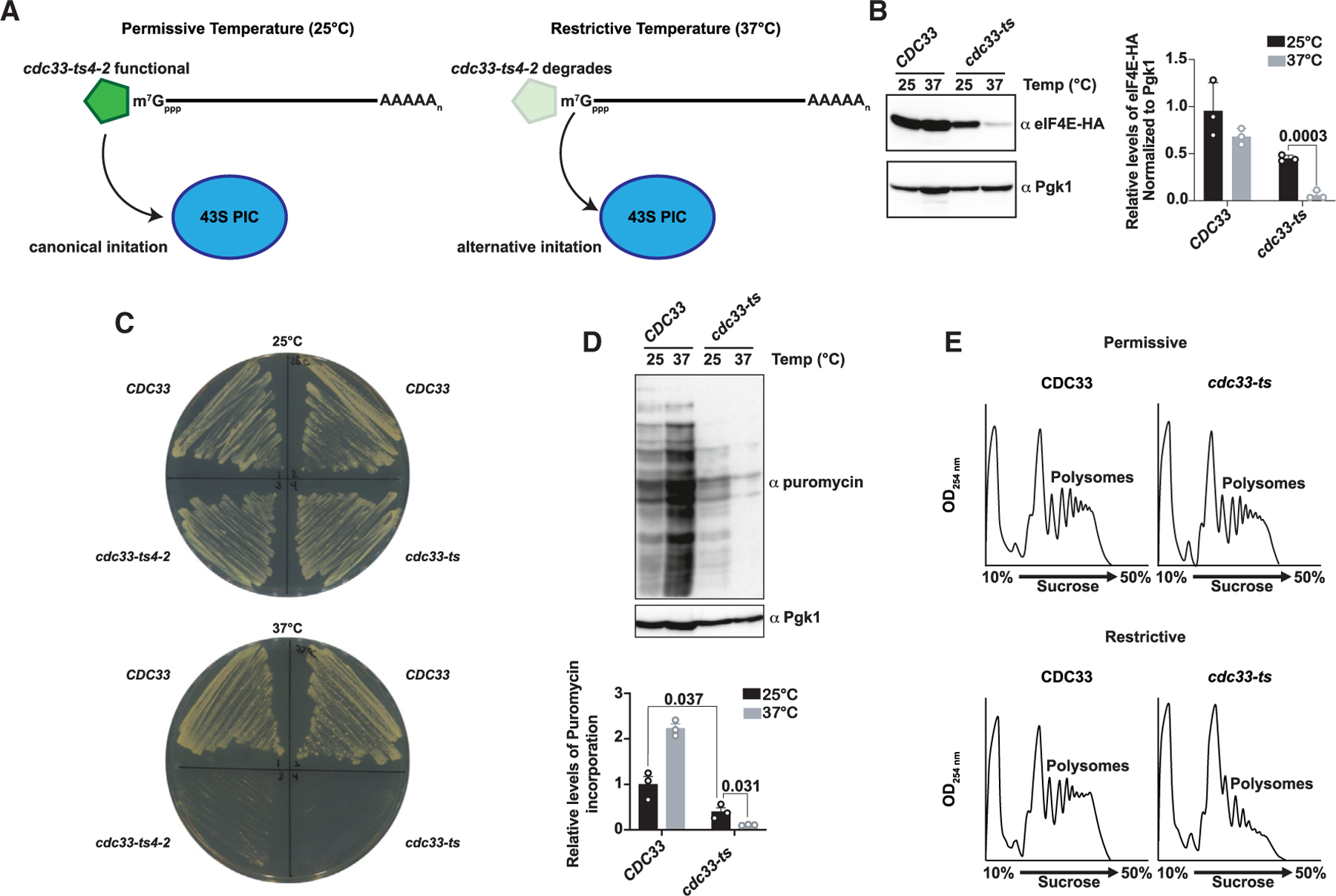
Translation is greatly diminished in cdc33-ts4-2 cells under restrictive conditions (A) Schematic of translation initiation under permissive (25°C) and restrictive (37°C) conditions in the *cdc33-ts4-2* strain. Under normal conditions, the cap-binding factor is stable and translation initiation proceeds as normal. Under restrictive conditions, the factor degrades, inhibiting canonical initiation. (B) Immunoblot analysis showing the levels of eIF4E in the *CDC33* and *cdc33-ts4-2* strains at 25°C and 37°C. The quantification of blots from three biological repeats is plotted on the right. The error bars represent the standard deviation around the mean. *p* values, which were determined using an unpaired parametric t test, are plotted above the values being compared. (C) *CDC33* and *cdc33-ts4-2* strains grown on YPD plates at 25°C and 37°C. The E73K and G179D mutations render the yeast temperature sensitive; shown is one plate of at least three replicates. (D) Immunoblot analysis of puromycin incorporation in the indicated cells at the depicted temperature. The quantification of blots from three biological repeats is plotted on the bottom. The error bars represent the standard deviation around the mean. *p* values, which were determined using an unpaired parametric t test, are shown above the values being compared. (E) Polysome profiles of whole-cell extracts from *CDC33* and *cdc33-ts4-2* cells under permissive and restrictive conditions. Cells were first grown at 25°C to OD ~ 0.5, then the culture was split in two, with half the culture shifted to the restrictive condition for an hour before both cultures were collected. Absorbance readings were taken continuously at OD_254 nm_.

To characterize translation events in yeast in the absence of cap recognition by eIF4E, we took advantage of a temperature-sensitive allele of the factor—*cdc33-ts4-2*.^33,34^ We chose this particular allele because, under permissive conditions (25°C), the factor is stable and functions properly but, under restrictive conditions (37°C), the factor is rapidly degraded^35,36^ (Figure 1A). To generate strains in the BY4741 background, we introduced the E73K and G179D mutations using an integrating plasmid targeting the native *CDC33* locus. Control strains were generated using the same plasmid, but instead bearing the wild-type sequence. Sequencing of the *CDC33* locus confirmed that the mutations were introduced as intended. We also generated an additional set of strains with eIF4E tagged at the C terminus with a hemagglutinin (HA)-tag, enabling us to measure its levels by immunoblotting. As expected, eIF4E levels were approximately 2-fold lower in the temperature-sensitive mutant under the permissive temperature, and further reduced to ~5% of wild-type levels when shifted to 37°C (Figure 1B). Plating assays also confirmed that the E73K and G179D mutations in eIF4E render yeast temperature sensitive (Figure 1C). Prior work characterizing the *cdc33-ts4-2* mutant showed significant inhibition in [^35^S] methionine incorporation compared with a wild-type strain when shifted to 37°C.^33^ To assess how the mutant factor affects translation in the BY4741 background, we conducted puromycin labeling of nascent peptides. Consistent with other findings,^37^ translation appears to be greater than an order of magnitude lower in the mutant at 37°C as compared with the wild-type strain (Figure 1D). We further conducted polysome profiling analysis of wild-type and mutant cells at 25°C and 37°C (Figure 1E). As expected, *CDC33* and *cdc33-ts4-2* cell profiles looked largely similar at 25°C. At 37°C, *cdc33-ts4-2* cells showed a significant loss of polysomes, whereas *CDC33* cells nearly completely retained their polysome levels. Interestingly, while polysomes were largely lost, some polysomes were still observed, suggesting that a small population of transcripts remain actively translated even under eIF4E-depleted conditions.

### Ribosome profiling of *CDC33* and *cdc33-ts4-2* cells

**Figure 2.**
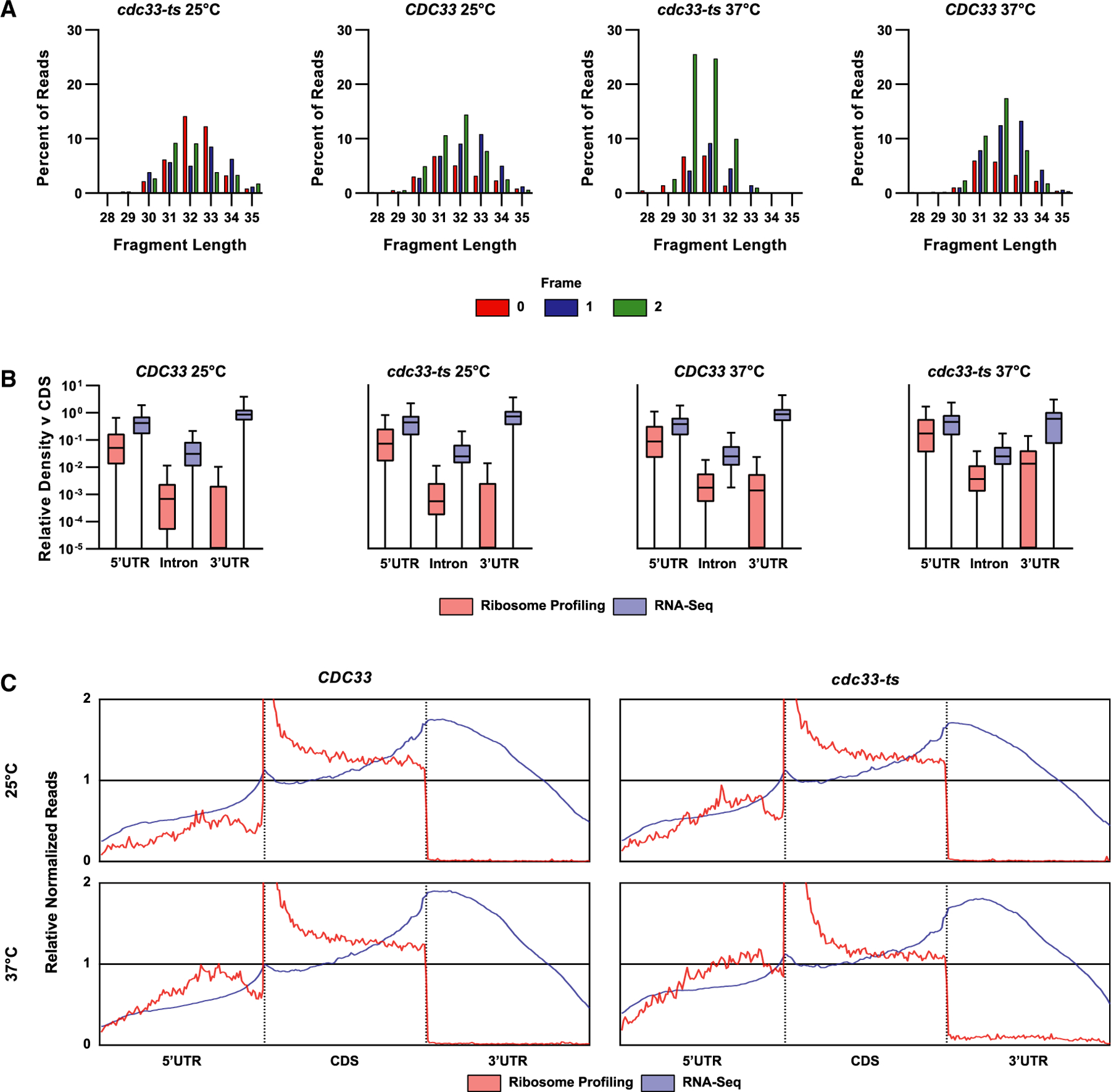
Ribosome-profiling analysis of CDC33 and cdc33-ts4-2 cells under permissive and restrictive temperatures (A) Bar graphs plotting the percent of reads that map to the indicated frame for the given fragment length. The frame for each read was assigned by offsetting from the 3′ end of the fragment with the offset for each fragment length, calculated based on the distance from the mapped 3′ end to the annotated start of the CDS. The plots here show one replicate of biological duplicates. (B) Box and whisker plots showing the relative density of reads mapping to the indicated feature, normalized by the feature length and the mean coverage of the associated CDS. Before plotting, outliers were removed using GraphPad Prism due to high variance in the distribution of densities, indicating outsized contribution from a small subset of genes. The plots show the average of biological duplicates for ribosome profiling and the average of biological triplicates for RNA-seq. (C) Plots displaying metagene analysis of ribosome profiling and RNA-seq reads. Coverage by ribosomes was calculated using the midpoint of the ribosome-protected fragments as a “pseudo A-site.” For RNA-seq reads, coverage across the entire mapped fragment was utilized. Coverage at each position in genes with 5′ UTR, CDS, and 3′ UTR of at least 100 nt in length were normalized by the mean coverage across the whole gene. Normalized coverages were then separated by the feature they mapped to, distributed evenly across 100 bins, and averaged across all analyzed genes. Relative normalized reads were plotted against a model gene 300 nt in length, with the first 100 nt representing the 5′ UTR and the last 100 nt representing the 3′ UTR. The dashed vertical lines indicate the start and stop of the CDS in the model gene, respectively.

To identify transcripts whose translation is resistant to eIF4E depletion, we conducted ribosome profiling^38^ on *CDC33* and *cdc33-ts4-2* cells subjected to both permissive and restrictive conditions. In parallel, we subjected the same cells to RNA sequencing (RNA-seq). One of the hallmarks of ribosome profiling is a distribution of fragments centered around 28 nt with an enrichment of in-frame reads. However, initial quality control of ribosome-profiling reads mapped to the genome did not show the typical distribution around 28 nt nor an enrichment of in-frame reads (Figure 2A), possibly due to incomplete RNase digestion. To confirm that our ribosome-profiling reads faithfully reflected ribosome-protected fragments and not free mRNAs, we conducted metagenomic analysis of both our ribosome profiling and RNA-seq data. Reassuringly, metagenomic analysis of our ribosome-profiling reads showed enrichment of reads mapping to gene coding sequences and 5′ UTRs, with minimal coverage of introns or 3′ UTRs (Figure 2B). Analysis of positional coverages also showed coverage centered around the annotated start of the coding sequence (CDS) Figure 2C), characteristic of ribosome-protected fragments.^38^

**Figure 3.**
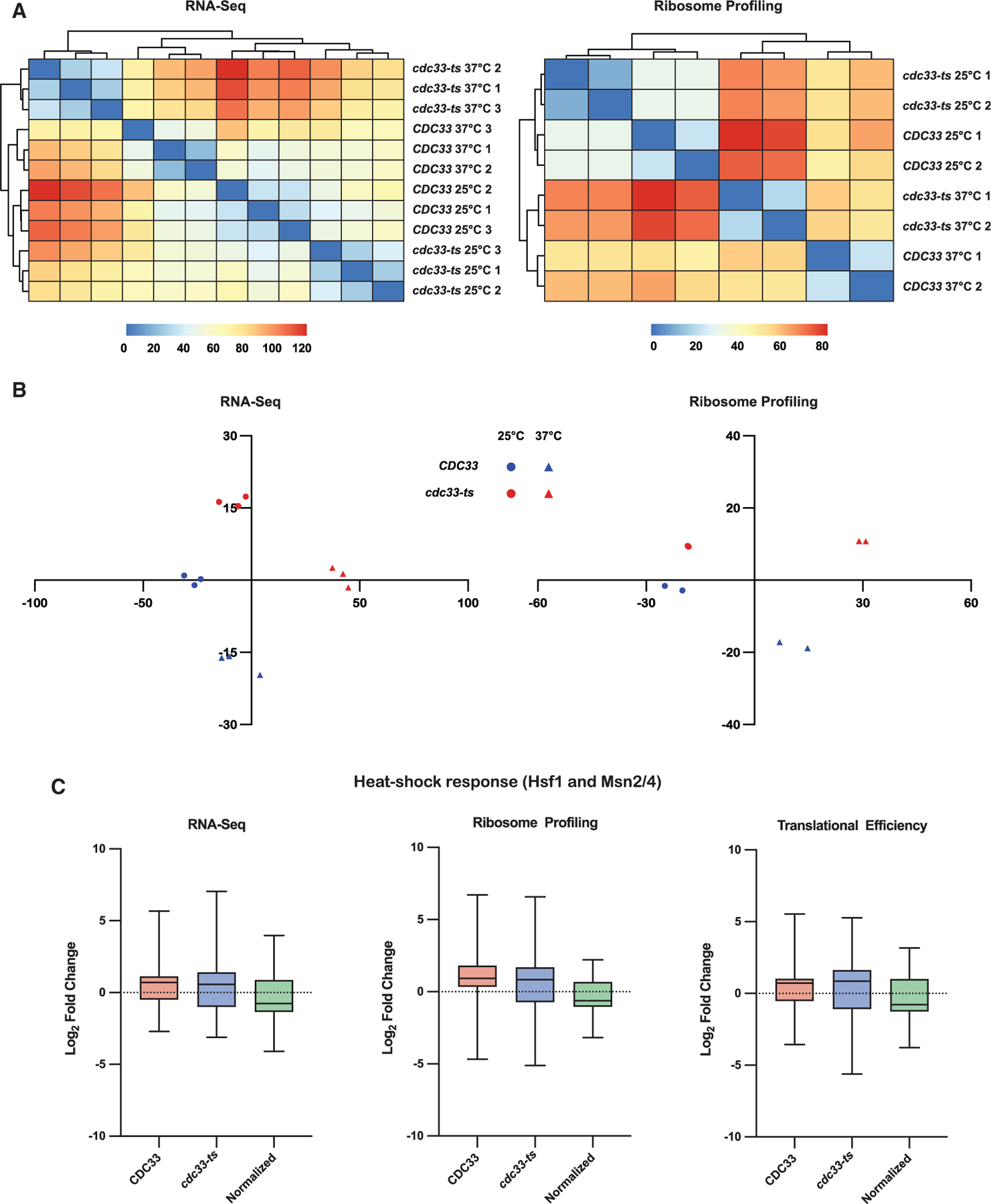
Differential gene expression analysis can account for changes due to heat shock (A) Heatmap and clustering of Euclidean distances for variance stabilized transformed count data for RNA-seq and ribosome profiling samples. (B) PCA plot of the transformed counts analyzed in (A). (C) Box and whisker plots of mean fold changes in mRNA expression (RNA-seq), ribosome occupancy (ribosome profiling), or ribosome occupancy normalized to mRNA levels (translational efficiency) for genes annotated as upregulated by Hsf1, Msn2, or Msn4 on Yeastract+. Only genes whose fold changes were marked as significant (adjusted *p ≤* 0.05) were plotted.

Confident that our ribosome-profiling results reflected true ribosome occupied fragments, we proceeded with differential gene analysis using the Salmon-DESeq2 pipeline.^39,40^ Although cycloheximide is known to introduce artifacts in ribosome-profiling data,^41–43^ gene-level analysis appears to be minimally biased (Figure S1). Quality control analysis of ribosome profiling and RNA-seq reads mapped to the transcriptome showed expected clustering of replicates, both by Euclidean distance and PCA (Figures 3A and 3B). Because our samples clustered closer by temperature than by strain (Figure 3A), we suspected that the majority of observed changes in gene expression were due to the heat shock response. However, pairwise comparison between *cdc33-ts4-2* and *CDC33* at 37°C, normalized to their counterparts at 25°C, was able to isolate changes in gene expression due to eIF4E depletion. Indeed, we observed that for genes upregulated by Hsf1 and Msn2/Msn4, the main regulators of the heat shock response,^44–46^ the expected increase in gene expression disappears as a result of the normalization procedure (Figure 3C).

**Figure 4.**
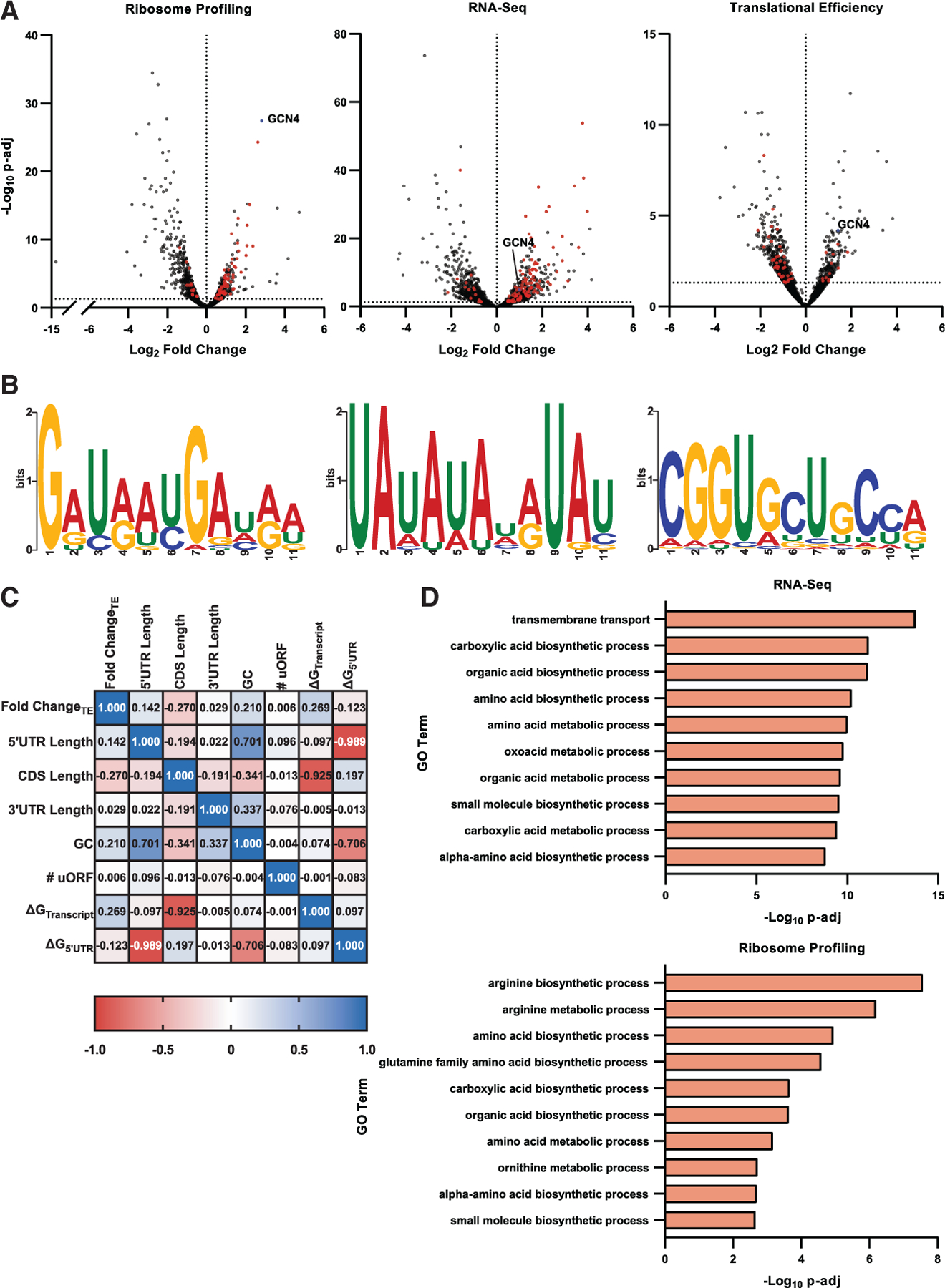
Loss of eIF4E leads to activation of the integrated stress response (A) Volcano plots of the fold change in mRNA expression (RNA-seq), ribosome occupancy (ribosome profiling), or ribosome occupancy normalized to mRNA levels (translational efficiency) plotted against the statistical significance of that change. Changes reflect changes in gene expression due to loss of eIF4E, for all genes passing automatic filtering in DESeq2. The vertical dashed line denotes a log_2_ fold change (LFC) of 0, while the horizontal dashed line denotes an adjusted *p* value of 0.05. Genes that belong to the Gcn4 regulon are marked in red, while *GCN4* is marked in blue. (B) Motifs found in genes upregulated due to loss of eIF4E (LFC ≥ 1) in the RNA-seq dataset, as determined by STREME from the MEME Suite software. No motifs passing statistical significance were found for genes that showed increased ribosome occupancy or translational efficiency. (C) Pearson correlation matrix of the indicated features measured against one another. Change_TE_ refers to the calculated differential translational efficiency values, as plotted in (A). (D) GO term search results for upregulated genes as a result of loss of eIF4E in the RNA-seq and ribosome profiling datasets. Searches were done using the Saccharomyces Genome Database (SGD) GO Term Finder tool on the 100 most upregulated genes (LFC ≥ 1) in each dataset. Displayed are the top 10 terms from each search.

Next, we plotted changes in transcript abundance, ribosome occupancy, and translational efficiency (TE); ribosome occupancy normalized to transcript abundance (Figure 4A). We then searched for motifs in those genes that had at least a 2-fold change in expression for each dataset. No motifs were detected as significant for upregulated genes in the ribosome profiling or TE datasets, but several motifs were detected for genes in the RNA-seq dataset. However, only the efficiency element necessary for polyadenylation of mRNAs stood out (Figure 4B).^47^ Further analysis of transcript features also did not yield any obvious grouping of upregulated genes; no strong correlation was observed between change in TE with 5′ UTR length, coding sequence length, 3′ UTR length, GC content, number of uORFs, or folding energy (Figure 4C). We pondered whether structural complexity of the 5′ UTR, denoted as ΔG_5′ UTR_ instead of simply the 5′ UTR length, was perhaps a better indicator of translation under eIF4E-depleted conditions. However, we also did not observe significant correlation between ΔG_5′ UTR_ and change in TE (Figure 4C). In addition, we explored the possibility that differences in TE might be driven by differences in distance to the consensus Kozak sequence. In yeast, the consensus sequence is 5′-(A/U)A(A/C)A(A/C)AAUGUC(U/C) and mutation of the purine at position −3 and/or the adenine at position −1 can significantly alter expression of the protein.^48–51^ However, motif analysis of the start codon context—both six nucleotides up- and downstream of the AUG of the 100 most upregulated and downregulated genes—failed to discover any deviation from the consensus Kozak sequence. Likewise, Gene Ontology (GO) analysis of genes failed to detect any process as significant for genes with increased TE.

### Depletion of eIF4E activates the ISR

**Figure 5.**
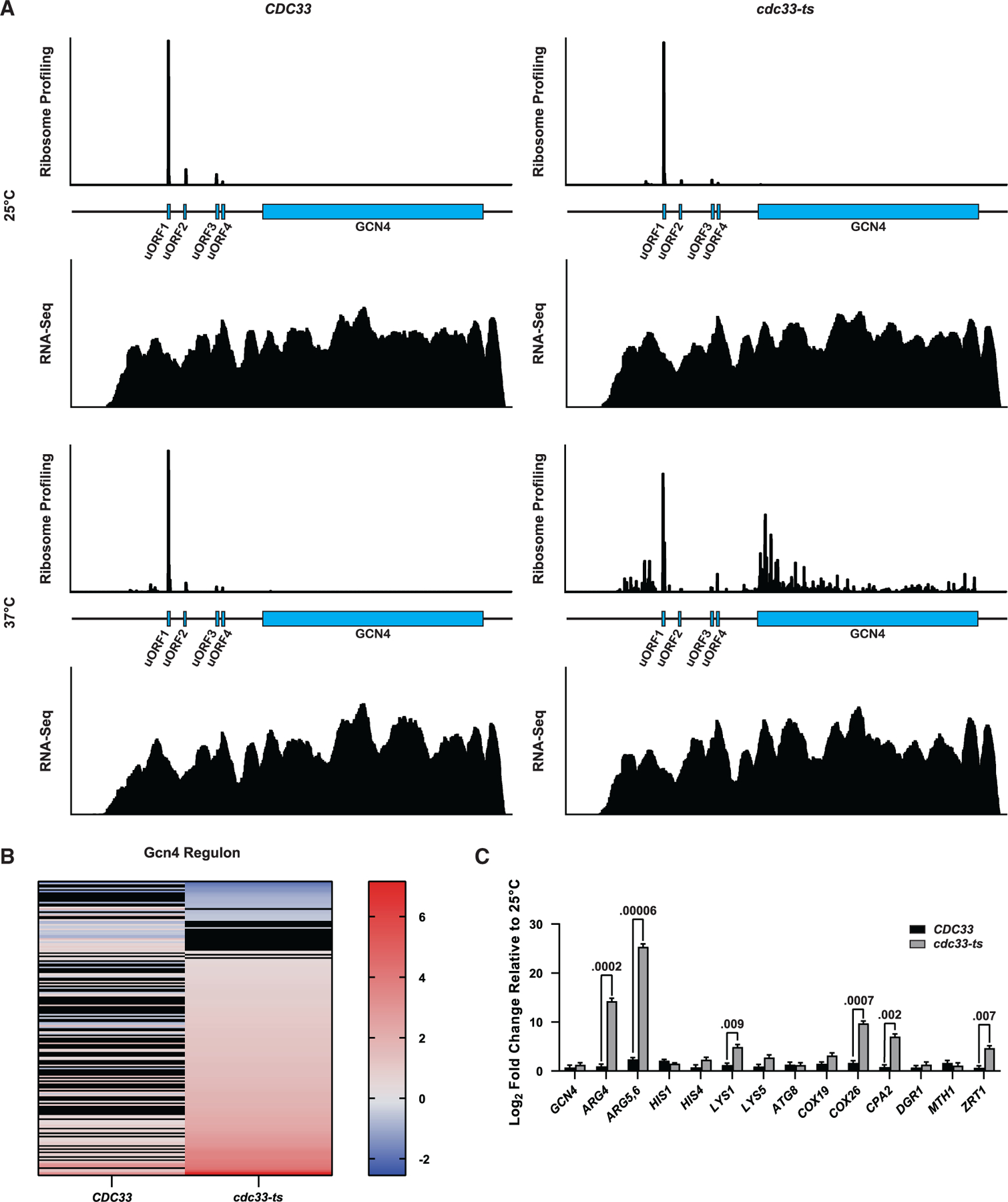
GCN4 is translated under eIF4E-depleted conditions (A) Ribosome occupancy and RNA-seq coverage plots of the *GCN4* transcript. Coverage by ribosomes was calculated by using the midpoint of the ribosome-protected fragments as a pseudo A-site. For RNA-seq reads, coverage across the entire mapped fragment was utilized. (B) Heatmap of log_2_ fold changes for genes belonging to the Gcn4 regulon in the RNA-seq dataset. Both strains at the restrictive condition were compared with the permissive condition. Rows colored in black indicate a fold change that did not have an adjusted *p ≤* 0.05. (C) RT-qPCR of the indicated genes in the *CDC33* and *cdc33-ts4-2* strains in the restrictive condition compared with the permissive condition. The expression of each gene was first normalized to expression of *TAF10*. Plotted are the average values of three biological replicates, with error bars representing the standard deviation around the mean. *p* values, which were determined using unpaired paramteric t test, are plotted above the values being compared.

Intriguingly, our analysis revealed *GCN4*, the key regulator of the ISR, as one of the top genes showing increased ribosome occupancy and TE as a result of the loss of eIF4E (Figures 4A and 4D). In support of these results, genome-mapped reads showed a significant increase in coverage of the main ORF of *GCN4* only in the *cdc33-ts4-2* strain at 37°C (Figure 5A). In agreement with increased de-repression of *GCN4* translation, RNA-seq analysis also showed that the Gcn4 regulon was significantly induced (Figure 5B). Moreover, GO analysis of RNA-seq and ribosome-profiling data showed the biological processes of amino acid synthesis to be significantly enriched in the mutant cells (Figure 4D). We validated our transcriptomic analysis by conducting RT-qPCR analysis of wild-type and mutant cells, which showed significant increases in expression of the Gcn4 regulon only in the mutant cells at 37°C (Figure 5C). These results are consistent with previous reports on the related *cdc33-1* mutant,^34^ where an amino acid starvation phenotype was observed under restrictive conditions.^34,52,53^

### *GCN4* translation is de-repressed via a non-canonical mechanism

**Figure 6.**
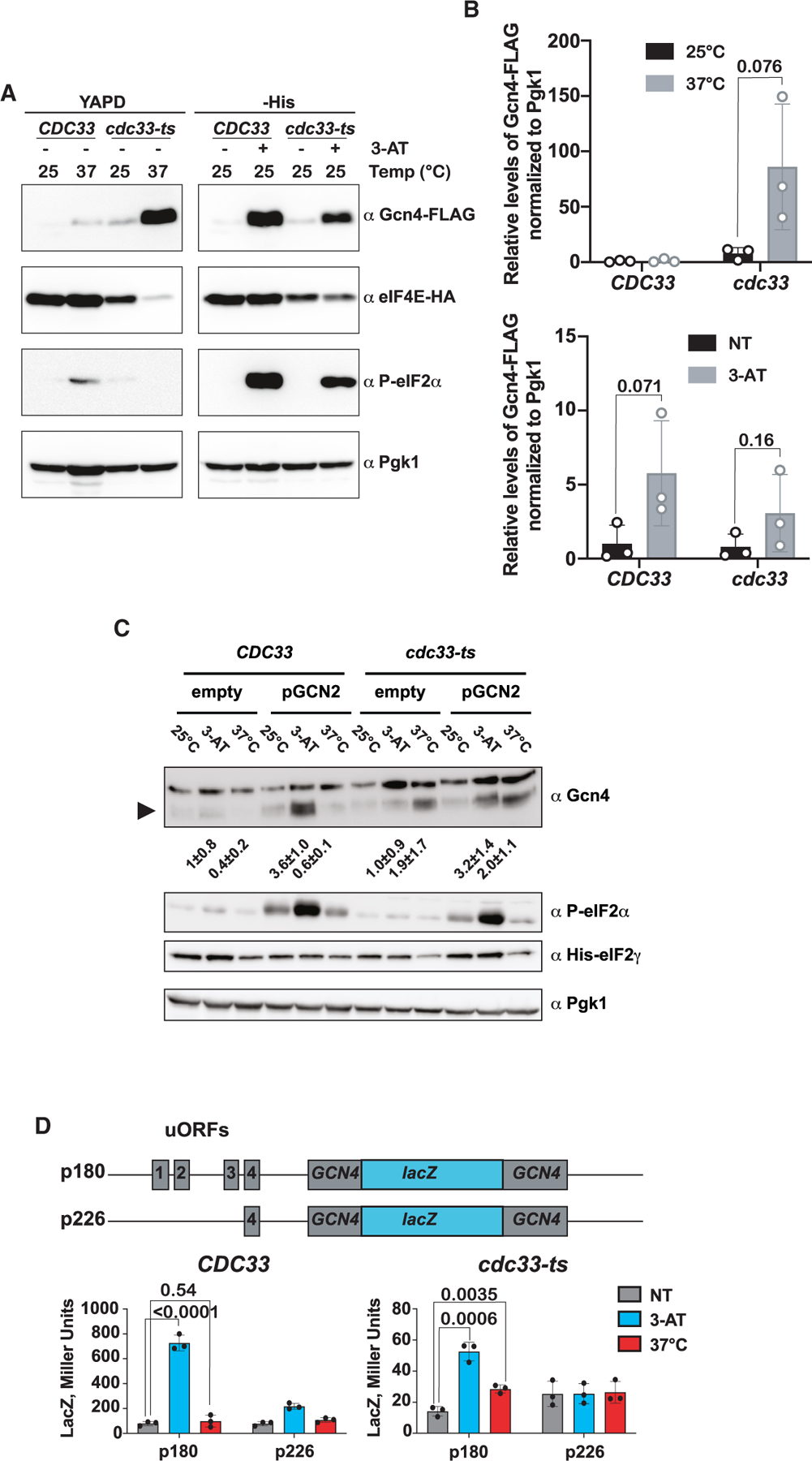
GCN4 translation is de-repressed without concordant eIF2α-phosphorylation or changes in ternary complex concentration (A) Representative immunoblots of whole-cell extracts collected from the indicated strains and conditions. (B) Bar graphs showing the quantification of immunoblots used to determine the relative levels of Gcn4 to Pgk1 in the labeled conditions. (C) Representative immunoblots used to follow the induction of Gcn4 in the indicated *gcn2Δ* backgrounds. The numbers below the Gcn4 blot represent the protein level of Gcn4 normalized to Pgk1 for each condition, relative to its corresponding no-treatment level from three biological replicates. (D) Top is a schematic of the *GCN4-lacZ* fusion reporters used to study the mechanism of *GCN4* translation de-repression. Bottom shows bar graphs summarizing *lacZ* expression from the indicated reporters in *CDC33* and *cdc33-ts4-2* cells grown at the indicated conditions. In all cases, the average values of three biological replicates are plotted, with error bars representing the standard deviation around the mean. *p* values, which were determined using an unpaired parametric t test, are plotted above the values being compared.

Given the translational mechanism by which *GCN4* is regulated, the most parsimonious explanation for de-repression of *GCN4* translation in our *cdc33-ts4-2* cells is that depletion of eIF4E leads to phosphorylation of eIF2α. To answer this question, we conducted immunoblot analysis of eIF2α-phosphorylation levels in wild-type and mutant cells at 25°C and 37°C. To our surprise, we did not observe increased eIF2α-phosphorylation in *cdc33-ts4-2* cells, indicating that de-repression of *GCN4* translation appeared to be the result of an alternative mechanism (Figures 6A and 6B). By contrast, canonical de-repression of *GCN4* translation via treatment with 3-aminotriazole (3-AT) was accompanied by increased eIF2α-phosphorylation in both backgrounds (Figures 6A and 6B). To provide further support for an eIF2α-phosphorylation-independent mechanism of *GCN4* translational control, we introduced the *cdc33-ts4-2* mutations into a *gcn2Δ* background.^54^ As expected, deletion of *GCN2* completely abrogated the accumulation of Gcn4 and phosphorylation of eIF2α in response to treatment with 3-AT in both *CDC33* and *cdc33-ts4-2* cells. Complementing *gcn2*Δ with a plasmid-borne gene restored responsiveness to 3-AT. By contrast, depletion of eIF4E resulted in increased Gcn4 levels without concordant eIF2α phosphorylation, even in the absence of Gcn2 (Figure 6C). Together, our data suggest that eIF4E depletion leads to de-repression of *GCN4* translation in a Gcn2-eIF2α-phosphorylation-independent manner.

### De-repression of *GCN4* translation under eIF4E-depletion conditions requires scanning and bypass of the inhibitory uORFs

The four uORFs found in the 5′ UTR of *GCN4* play an important role in regulating its translation. Elimination of these elements results in elevated translation of the factor, regardless of the stress status of the cell.^55^ Interestingly, uORF1 and uORF4 are almost completely responsible for *GCN4* translational regulation.^14^ After translation of uORF1, ~50% of ribosomes are able to remain bound to the transcript after termination, resume scanning, and initiate again on a downstream ORF in a process known as reinitiation.^55^ Conversely, during translation of uORF4, ribosome dissociation is extremely efficient post termination and <1% of ribosomes remain to reinitiate on the *GCN4* main ORF.^55^ Under normal conditions, when eIF2α is not phosphorylated and TC levels are abundant, ribosomes that resume scanning after translation of uORF1 are able to bind another TC and reinitiate on uORF4, ultimately resulting in their dissociation and repression of *GCN4* translation. However, under stress conditions, when eIF2α is phosphorylated and TC levels are reduced, significantly more ribosomes are unable to bind a TC in time to reinitiate on uORF4; these subsequently bypass the inhibitory effect of uORF4 and translate *GCN4* main ORF.

We took advantage of *GCN4-lacZ* fusion reporters (Figure 6D; key resources table) generated by the Hinnebusch group^56–58^ to determine whether the de-repression of *GCN4* translation that we observe when eIF4E levels are depleted utilizes a similar mechanism. As expected, expression of the p180 reporter, which recapitulates the entire *GCN4* regulatory unit, is de-repressed in the presence of 3-AT in both the wild-type and temperature-sensitive mutant. Although the basal expression for the reporter in *cdc33-ts4-2* cells was significantly lower than what we measured in the wild-type strain at 25°C (Figure 6D), we measured an approximately 2-fold increase in the reporter in *cdc33-ts4-2* cells, which was not observed in *CDC33* cells, upon shifting to 37°C (Figure 6D). To test whether induction of the reporter depends on ribosome scanning, we utilized the p226 reporter, which has only the inhibitory uORF4. Expression of this reporter was not induced in *cdc33-ts4-2* cells at 37°C, suggesting that ribosome scanning is important for *GCN4* translation under reduced eIF4E levels. Consistent with this proposal, using reporters with stem loops introduced in the 5′ UTR^55^ to impede ribosome scanning inhibited induction of *lacZ* under both amino acid starvation and eIF4E-depleted conditions (Figures S2A and S2B).

Our findings suggest that the 5′ UTR does not support internal initiation by the ribosome when cap recognition is inhibited. To add further support for this conclusion, we constructed a polycistronic dual luciferase reporter with a Renilla luciferase and firefly luciferase separated by a stop codon. We inserted the 5′ UTR of *GCN4*, with or without the first 20 codons of the main GCN4 ORF, between the two genes (Figure S2C). As expected, the ratio of firefly to Renilla luminescence was ~1% for the polycistronic reporter relative to the ratio measured for the translation fusion reporter at 25°C and 37°C. Introducing the 5′ UTR of *GCN4* had no detectable effect on firefly luminescence, suggesting that it cannot drive internal initiation in the absence of eIF4E (Figure S2D).

### TC concentration does not appear to be altered in *cdc33-ts4-2* cells under restrictive conditions

Although the standard model for de-repression of *GCN4* translation is through phosphorylation of eIF2α, ultimately any mechanism that depletes TC levels would also result in de-repression. A previous report on *cdc33-1* cells showed a slight reduction in initiator methionyl tRNA and eIF2 subunits, including eIF2γ, under restrictive conditions.^59^ If loss of eIF2γ levels is indeed the responsible mechanism, then overexpression of eIF2γ should inhibit the de-repression of *GCN4* translation. To test this hypothesis, we overexpressed eIF2γ in our *cdc33-ts4-2* cells. However, overexpression of eIF2γ did not inhibit the de-repression of *GCN4* translation (Figure S3A) but instead appeared to further de-repress GCN4 translation, similar to observations in wild-type cells overexpressing eIF2γ.^60^ To approach rescue of eIF2γ levels in an orthogonal manner, we overexpressed Cdc123 in the same background. Cdc123 is an upstream factor responsible for eIF2γ maturation and proper TC formation.^61,62^ Notably, however, overexpression of Cdc123 had no detectable effect on Gcn4 accumulation when eIF4E levels were depleted (Figure S3B). Taken together, our observations suggest that depletion of eIF4E does not significantly alter eIF2γ levels such as to contribute to de-repression of *GCN4* translation.

Another possibility was that depletion of eIF4E may indirectly impact tRNA levels, which would ultimately lead to a reduction in TC levels. To test this possibility, we isolated total RNA from wild-type and mutant cells at 25°C and 37°C and conducted northern blot analysis to assess the level of several tRNAs. We found that depletion of eIF4E levels did not significantly alter the relative concentrations of all tRNAs tested (tRNA_iMet_, tRNA_eMet_, tRNA_Arg_, and tRNA_His_) (Figure S4). Collectively, our data suggest that the observed de-repression of *GCN4* translation under eIF4E-depleted conditions occurs via a mechanism distinct to eIF2α phosphorylation or changes in TC concentration.

### eIF4A levels are important for de-repression of *GCN4* translation

**Figure 7.**
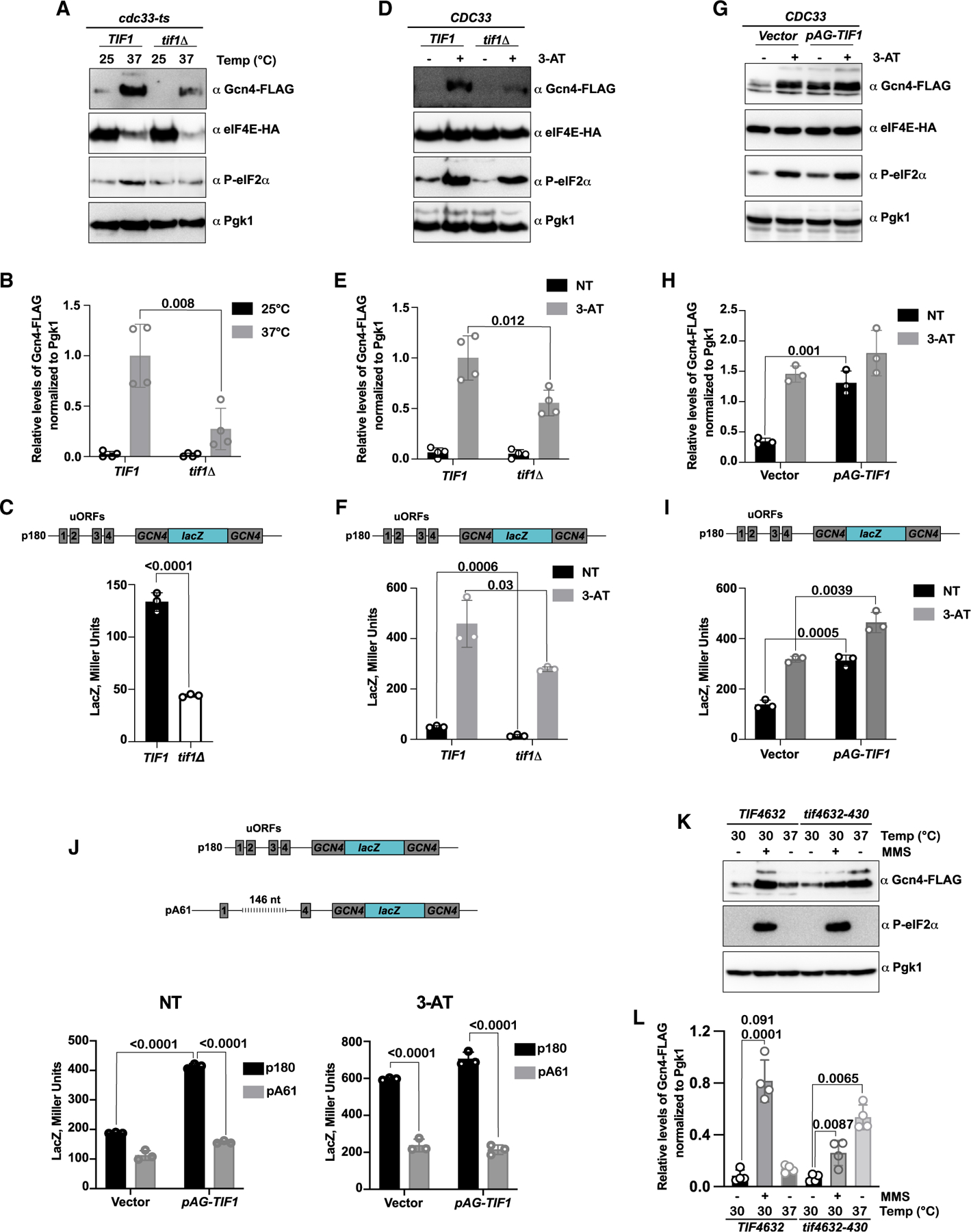
eIF4A levels contribute to GCN4 translation de-repression (A) Representative immunoblots used to follow the relative levels of Gcn4-FLAG, eIF4E-HA, P-eIF2α, and Pgk1 levels in *cdc33-ts4-2* cells grown at 25°C and 37°C. (B) Bar graphs summarizing the quantification of four immunoblots used to show the relative levels of Gcn4 in the indicated strains and conditions. (C) Bar graph showing the *lacZ* expression from the p180 plasmid (*GCN4-lacZ* fusion) in the presence and absence of *TIF1*. (D) Representative immunoblot used to analyze the Gcn4 levels in wild-type and *TIF1Δ* cells in the absence and presence of 3-AT. (E) Bar graphs summarizing the quantification data for relative Gcn4 levels to Pgk1 from four immunoblots (similar to the one shown in D). (F) Similar to (C), but used to assess *lacZ* expression in response to addition of 3-AT in wild-type and *TIF1Δ* cells. (G) Representative immunoblot used to analyze the relative levels of Gcn4 in wild-type cells harboring an empty vector or a *TIF1* plasmid in the absence and presence of 3-AT. (H) Bar graphs depicting the relative levels of Gcn4 to Pgk1, under the indicated conditions, from three independent biological replicates. (I) Bar graphs showing *lacZ* expression from the indicated reporter in wild-type cells harboring the indicated plasmids in the absence and presence of 3-AT. (J) Top shows a schematic comparing the p180 reporter to the pA61 one. Bottom shows bar graphs summarizing *LacZ* expression from the indicated reporters in cells transformed with the indicated plasmid in the absence (NT) or presence of 3-AT. (K) Representative immunoblot used to compare the levels of Gcn4 in the indicated cells. (L) Bar graph describing the quantification of the relative levels of Gcn4 to Pgk1 under the indicated conditions. In all cases, the error bars represent the standard deviations around the mean. *p* values, which were determined using an unpaired parametric t test, are plotted above the values being compared.

Another important element of *GCN4* translational control is the distance between uORF1 and uORF4, which has been tuned to prevent ribosomes from bypassing uORF4 unless conditions necessitate such.^14^ Thus, mechanisms that can decrease transit time between the two uORFs by increasing scanning speed should also result in de-repression of *GCN4* translation. eIF4E is a core component of the eIF4F complex, which, in addition to mRNA recruitment, also promotes ribosome scanning. eIF4A, another eIF4F-component, is a DEAD-box helicase that assists the small subunit during scanning by unwinding mRNA secondary structures.^6^ eIF4E is substoichiometric to eIF4A,^63^ and the disparity between their concentrations is exacerbated in *cdc33-ts4-2* cells under restrictive conditions (Figure 1B). Therefore, we hypothesized that when eIF4E levels are depleted, the local concentration of eIF4A on a particular eIF4E-bound mRNA would dramatically increase. This, in turn, might increase ribosome scanning speed, enabling ribosomes to bypass uORF4 after translating uORF1, even without a reduction in TC levels. To test this hypothesis, we deleted *TIF1*, one of the two paralogs that encode eIF4A, in our wild-type and eIF4E-mutant cells. Deletion of *TIF1* suppressed de-repression of *GCN4* translation in *cdc33-ts4-2* cells at 37°C (Figures 7A and 7B). Further confirming our observations, reducing eIF4A concentration resulted in an approximately 3-fold reduction in *lacZ* expression from a *GCN4-lacZ* fusion reporter (Figure 7C). Similarly, deleting *TIF1* in wild-type cells suppressed 3-AT-induced de-repression of *GCN4* translation (Figures 7D–7F). To rule out the possibility that the deletion of *TIF1* leads to global reduction in translation, we conducted puromycin labeling of nascent peptides. The results of these experiments, which are shown in Figure S5, show no significant differences between wild-type and *tif1Δ* cells in puromycin reactivity with elongating ribosomes.

Our model—that the relative concentration of eIF4A to eIF4E can dramatically alter the activation of the ISR—predicts that overexpression of eIF4A would lead to de-repression of *GCN4* translation, even under non-starved conditions. To test this prediction, we generated a high-copy plasmid harboring the *TIF1* gene, together with its own promoter and UTRs. We then introduced this plasmid, along with an empty-vector control, into wild-type cells and assessed for the accumulation of Gcn4 in the absence and presence of 3-AT (Figure 7G). In complete agreement with our model, we measured a more than 3-fold increase in the levels of Gcn4 when eIF4A was overexpressed under non-starved conditions (Figures 7G and 7H). Remarkably, the levels of Gcn4 in the presence of increased eIF4A levels were comparable to those observed in the presence of 3-AT (Figures 7G and 7H), suggesting that this de-repression mechanism is as effective as the canonical one. Additionally, this accumulation of Gcn4 was not accompanied by alteration to eIF2α phosphorylation status (Figures 7G and S6). We further complemented these assays with our *GCN4-lacZ* fusion reporter. Consistent with our immunoblot assays, we observe a more than 2-fold increase in the expression of the reporter when eIF4A is overexpressed, similar to the increase induced by the addition of 3-AT (Figure 7I). Thus, eIF4A, like eIF4E, appears to play an important role during de-repression of *GCN4* translation. However, in contrast to eIF4E, increasing eIF4A levels appears to further de-repress *GCN4* translation.

To add further support to our model, we utilized a *GCN4-lacZ* reporter, pA61, with an increased distance between uORF1 and uORF4 by 146 nucleotides.^56^ If our model is correct, then increasing the inter-ORF distance should suppress the effect of eIF4A overexpression by giving more time for small subunits to acquire a new TC and reinitiate on uORF4. As expected, overexpression of eIF4A did not result in *lacZ* induction from the pA61 reporter (Figure 7J). The finding that increased distance between uORF1 and uORF4 leads to complete loss of eIF4A-mediated induction of the *GCN4* reporter suggests that increased local concentration of the factor leads to bypassing the inhibitory effect of uORF4 by the ribosome during translation of *GCN4*.

### Similar to eIF4E, depletion of eIF4G results in accumulation of Gcn4

So far, our analysis has focused on the interplay between eIF4E and eIF4A during *GCN4* translation and has not scrutinized a potential role for the other member of the eIF4F complex, eIF4G. Because eIF4G is a scaffold protein directly involved in bridging eIF4E and eIF4A on mRNAs, we hypothesized that its inhibition would also result in increased local concentration of the helicase on activated mRNAs. Interestingly, in a previous study, inhibition of eIF4G was found to increase translation of a *GCN4* reporter, but it was suggested that this was due to enhanced reinitiation.^64^ These studies, however, did not assess the effect of eIF4G on endogenous Gcn4 levels. In yeast, eIF4G is encoded by two paralogs (*TIF6321* and *TIF4632*). We took advantage of a yeast strain in which both paralogs are deleted, with eIF4G supplemented on a plasmid carrying the *TIF4632* gene, as well as a derivative strain where the wild-type eIF4G plasmid is replaced by the temperature-sensitive allele *tif4632-430*. This mutation inhibits the interaction between eIF4G and eIF4E at 37°C.^64,65^ To determine how the inhibition of this interaction impacts *GCN4* translation, we added a C-terminal FLAG tag to the endogenous *GCN4* gene in both wild-type and mutant strains.

Here, we treated cells with methyl methane sulfonate (MMS) because alkylation stress is known to robustly activate Gcn2.^66^ As expected, we observe significant Gcn4 accumulation, concomitant with eIF2α phosphorylation, upon addition of MMS in both strains (Figures 7K and 7L). By contrast, we only observed accumulation of Gcn4 in the *tif4632-430* cells at 37°C, without any increase in eIF2α phosphorylation. Therefore, it appears that inhibition of eIF4G can also de-repress *GCN4* translation via an eIF2α phosphorylation-independent mechanism, similar to what we observe for eIF4E depletion. Altogether, our data point out to a potential unusual mechanism for how alteration to relative levels of the eIF4F components drives the translation of *GCN4* and activation of the ISR independent of eIF2α phosphorylation.

### Overexpression of the eIF4E-BP Caf20 de-represses translation of *GCN4*

Our findings suggest that, by regulating eIF4E levels, cells can de-repress translation of *GCN4* without having to phosphorylate eIF2α. Naturally, we were curious to see whether inhibition of the TOR pathway, which results in the dephosphorylation of eIF4E-BPs and subsequent sequestration of eIF4E, would recapitulate our observations. Such an experiment, however, is complicated by the observation that TOR inhibition in yeast also activates Gcn2.^67^ To circumvent this pleiotropic effect of TOR inhibition, we decided to directly overexpress Caf20, one of the eIF4E-BPs in yeast.^68^ We generated a constitutively dephosphorylated Caf20 mutant (S91A and T102A) to avoid potential phosphorylation and subsequent inactivation by TORC1 signaling. Notably, in *CDC33* cells, overexpression of the factor at 25°C had little to no effect on Gcn4 levels (Figure S7). We hypothesized that this could be due to eIF4E being in excess of Caf20, given that we were unable to observe a phenotype on translation. In accordance with our hypothesis, overexpression of Caf20 in *cdc33-ts4-2* cells at 25°C, where eIF4E levels are ~30% of levels in *CDC33* cells, resulted in a dramatic increase of Gcn4 levels (Figure S7). Surprisingly, Gcn4 levels increased in both Caf20 overexpression strains at 37°C (Figure S7). Given that eIF4E levels are largely unchanged in the wild-type background at elevated temperatures, it is unclear how overexpression of Caf20 results in de-repression of *GCN4* translation. Overall, our observations suggest a potential link between the TOR and ISR pathways, independent of eIF2α phosphorylation, by which sequestration of eIF4E by its binding proteins as a result of TOR signaling can trigger de-repression of *GCN4* translation.

## DISCUSSION

In eukaryotes, canonical initiation requires the coordinated effort of a multitude of initiation factors. These factors recruit the small ribosomal subunit to the 5′ cap of the mRNA and aid in identifying the correct start codon.^3,4,69^ On the other hand, while canonical initiation is responsible for translating the majority of mRNAs, evidence increasingly supports the notion that cap-independent mechanisms play critical roles in health and disease.^70^ Because many of the mechanisms appear to be highly conserved and necessary for cell survival under stress conditions, much effort has been directed at trying to elucidate their function and usage, particularly in the case of diseases with dysregulated translation, such as autoimmune diseases, neurodegeneration, and cancer.^71,72^ However, many studies have limited their focus to specific transcripts and the features that allow them to evade cap dependence during initiation.^73,74^ The few studies that have attempted to define the global landscape of cap-independent translation have not used unbiased and systematic approaches, precluding the ability to glean important data about biologically relevant processes.^75^ To circumvent these issues, we used a temperature-sensitive allele of eIF4E to systematically disrupt cap recognition in yeast in an unbiased manner. We confirmed that, under the restrictive temperature, the mutant allele leads to a significant reduction in translation, as judged by puromycin labeling of nascent peptide and ribosome profiling (Figures 1D and 1E). In order to identify transcripts whose translation is resistant to eIF4E loss, we subjected wild-type and mutant cells under permissive and restrictive conditions to ribosome profiling. Even though we found that the translation of several transcripts was resistant to the loss of eIF4E (Figure 4A), the mechanisms responsible were not readily discernable; we did not observe any obvious correlations between translation efficiencies and CDS length, UTR length, structure, GC content, or number of uORFs (Figure 4C). As detailed below, however, our data suggest that some mRNAs, whose translation has been described as cap insensitive, are instead sensitive to the relative concentration of the eIF4F factors, particularly the ratio of eIF4A to eIF4E.

Interestingly, our ribosome-profiling studies revealed that the loss of eIF4E leads to increased translation of the *GCN4* main ORF, with an almost 8-fold increase in ribosome occupancy in the mutant strain at the restrictive temperature (37°C) compared with the wild-type strain (Figure 4A). This was consistent with our transcriptomic and RT-qPCR analyses, which showed induction of the Gcn4 regulon (Figures 5B and 5C). Furthermore, in agreement with the ribosome-profiling data, immunoblot analysis revealed that Gcn4 levels increased only in the mutant strain at 37°C (Figures 6A and 6B).

As described earlier, *GCN4* is primarily regulated at the translational level via a unique mechanism that takes advantage of four uORFs found in its 5′ leader sequence. To the best of our knowledge, the only mechanism that has been described for how cells drive the translation of *GCN4* has been through phosphorylation of eIF2α. As a result, we were surprised to observe no accumulation of phosphorylated eIF2α in the *cdc33-ts4-2* mutant at 37°C (Figure 6A). Given the lack of eIF2α phosphorylation, the simplest explanation for observed de-repression of *GCN4* translation is that depletion of eIF4E also leads to depletion of TC. However, neither direct overexpression of eIF2γ nor overexpression of the eIF2 assembly factor Cdc123—which regulates eIF2 levels^62,76^—restored translational control of *GCN4* (Figures S3A and S3B). We further showed that eIF4E depletion has little to no effect on the initiator tRNA and four other tRNA levels (Figures S4A and S4B). Alternatively, our findings can be readily rationalized by a model whereby eIF4E has a relatively higher affinity for the *GCN4* 5′ UTR compared with other transcripts. Thus, when the concentration of eIF4E is severely limited, activation of the *GCN4* transcript by the eIF4F complex would not be impacted as severely as other mRNAs. However, under this model, we would expect the ribosome occupancy of the *GCN4* main ORF, relative to the occupancy on the uORFs, to remain unchanged. In disagreement with this prediction, our ribosome profiling revealed that ribosome occupancy on the main ORF increased significantly relative to occupancy on the uORFs, suggestive of de-repression rather than overall increased recruitment (Figure 5A).

The eIF4F complex is required to activate mRNAs and recruit them to the small subunit. The core components of the complex—eIF4E, eIF4G, and eIF4A—each fulfill a distinct function during translation. Therefore, alteration to the levels of these factors is likely to have an impact on ribosome dynamics during initiation. Although the concentration of eIF4G and eIF4E are similar, the concentration of eIF4A is about 10-fold higher.^63^ This disparity is further exacerbated in the *cdc33-ts4-2* strain at 37°C, and somehow enables some small subunits to escape reinitiation on uORF4 and instead initiate on the main ORF of *GCN4*. Although ribosome recruitment to mRNAs in the *cdc33-ts4-2* strain is severely inhibited due to decreased eIF4E levels, once recruited to the *GCN4* transcript, it is possible that ribosome scanning will be faster due to increased local eIF4A concentration. Increased scanning speeds would enable more small subunits to bypass uORF4 before engaging a new TC. In agreement with this model, decreasing the concentration of eIF4A by deleting one of its paralogs, *TIF1*, reduced accumulation of Gcn4 under both amino acid starvation and eIF4E-depleted conditions (Figures 7A–7F). By contrast, overexpression of eIF4A resulted in increased derepression of *GCN4* translation under both repressive and derepressive conditions (Figures 7G–7I). Although a recent study has argued that eIF4A levels have no direct role in the scanning speed of the small subunit on a model mRNA *in vitro*,^77^ it is likely that the conformation of the small subunits that resume scanning on the 5′ UTR of *GCN4* differs from those engaged in canonical scanning. Indeed, increasing the distance between uORF1 and uORF4 of *GCN4*, and hence giving the small subunit more time to bind a TC, inhibited eIF4A-mediated *GCN4* translation de-repression (Figure 7J).

Although the above model of increased scanning by the small subunit under eIF4E depletion is appealing, the details by which this might occur have several caveats. Namely, because yeast eIF4G harbors only one eIF4A binding site,^78,79^ it is unclear how changing relative eIF4A concentrations could alter scanning dynamics. Notably, Yourik and colleagues suggested that, given that eIF4A is in vast excess of ribosomes, the protein coats mRNAs nonspecifically, keeping them partially unwound.^80^ As the ribosome traverses the transcript, the encounter between eIF4G and mRNA-bound eIF4A results in ATP hydrolysis and the threading of the RNA into the entry channel of the scanning 43S PIC. ADP-bound eIF4A then dissociates, allowing the small subunit to find the next eIF4A molecule and load the next segment of the RNA. This model may explain how the abundance of eIF4A is correlated with scanning speed of the ribosome. Regardless of the mechanism by which the relative concentrations of eIF4A to eIF4E and/or eIF4G contribute to *GCN4* translational control, our findings suggest that the eIF4F complex dynamics alter ribosome dynamics and reprogram gene expression.

In a broader sense, our observation that *GCN4* translation is de-repressed under conditions of eIF4E depletion provides a potential connection between TOR signaling and the ISR. Under amino acid starvation, eIF2α phosphorylation by Gcn2 lowers available TC levels, enabling scanning 40S subunits to reinitiate on the main ORF of *GCN4*. At the same time, amino acid starvation also inhibits TOR, leading to sequestration of eIF4E by eIF4E-binding proteins and inhibition of canonical cap recognition by 43S PICs.^81^ Both these processes respond to nutrient availability and, as a result, must coordinate their efforts during reprogramming of gene expression.^21^ If depletion of eIF4E does indeed lead to increased scanning speed by the ribosome on the *GCN4* mRNA, this would provide a potential mechanism by which the two pathways are interconnected. Such a mechanism might have evolved to provide redundancy between the two pathways. Should Gcn2 fail to become activated or eIF2α fail to be phosphorylated, signaling from the TOR pathway can activate the ISR instead. Conversely, if the TOR pathway fails to recognize an amino acid deficiency, the cell has an alternative sensor in the form of Gcn2, with activation of the ISR driving restoration of homeostasis and reengagement of proper TOR pathway functioning. Curiously, studies in human cell culture showed that mTOR activation, and not its inhibition, can lead to ATF4 translation.^82–84^ In contrast, in yeast, inhibition of TORC1 by rapamycin leads to *GCN4* translation, albeit in a Gcn2-mediated and eIF2α phosphorylation-dependent manner.^67,85^ Notably, we were able to bypass this interdependence between the two pathways by overexpressing an eIF4E-BP and found it to result in increased Gcn4 levels (Figure S7), albeit under conditions when eIF4E levels are slightly reduced. Further investigations are clearly needed to elucidate the mechanisms that enable a unified response by these two pathways to nutrient availability in the environment.

Finally, our findings have important ramifications for the role of the eIF4F complex, and eIF4A in particular, in tumor biology. Emerging from several studies is the observation that overexpression of eIF4F subunits leads to tumorigenesis.^86–88^ Notably, most tumors display pronounced overexpression of eIF4A relative to eIF4E. Although eIF4A is overexpressed ~15-fold on average, eIF4E levels are not significantly changed (1.28-fold) in human primary cancers.^89^ As such, selective inhibition of eIF4A has recently surfaced as a possible anti-cancer therapy, including receptor-kinase-driven tumors.^90^ Interestingly, ATF4 (Gcn4’s homolog in mammals) is also overexpressed in tumors, where it appears to play important role in regulating proliferation, autophagy, metastasis, and drug resistance.^91–94^ Therefore, it is tempting to surmise that the skewed ratio of eIF4A to eIF4E in some of these tumors is responsible for the increased levels of ATF4. A better understanding of the relative concentration of eIF4E to eIF4A in translation dynamics would further our understanding of how cells regulate translation initiation for selective gene expression.

### Limitations of the study

Our work here uncovered a previously unappreciated role for how the relative ratio of the components of a core translation initiation complex can result in reprogramming of gene expression. We show that the relative concentration of eIF4E to eIF4A plays an important role during de-repression of *GCN4* translation in *Saccharomyces cerevisiae*. Our analysis was limited to assessing the contribution of eIF4F components on induction of the ISR. It is feasible that alteration of eIF4E levels may affect the levels of other initiation factors. These changes may confound our proposed model of how changes to the eIF4F complex alter initiation dynamics. More studies are needed to characterize the potential contributions of other initiation factors to *GCN4* translational control under eIF4E-depleted conditions, as well as the effects of eIF4E-depletion on their levels. Another limitation of our work is that we only used genetic manipulations to impair the eIF4F complex because many of the compounds that inhibit eIF4A helicase activity or the interaction between eIF4E and eIF4G show limited activity in yeast. As a result, it would be interesting to expand these studies into mammalian cells and ask how inhibition of eIF4A—or the interaction between eIF4E and eIF4G—impact ATF4 translation. These types of assays could provide insights into the translational landscape of cancer cell lines that exhibit altered ratios of the eIF4F components.

## STAR★METHODS

### RESOURCE AVAILABILITY

#### Lead contact

Requests and information for reagents and resources will be filled by Dr. Hani Zaher (hzaher@wustl.edu)

#### Materials availability

All stable reagents from this study are available from the lead contact.

#### Data and code availability

- RNA-seq and ribosome profiling data have been deposited at GEO and are publicly available as of the date of publication. Accession numbers are listed in the key resources table. Original western blot images have been deposited at Mendeley Data: https://doi.org/10.17632/gg5zwy34bc.1 and are publicly available as of the date of publication. The DOI is listed in the key resources table.
- Code used to analyze ribosome profiling and RNA-seq data has been deposited at Zenodo: https://doi.org/10.5281/zenodo.7617427 and is publicly available as of the date of publication. DOIs are listed in the key resources table.
- Any additional information required to reanalyze the data reported in this paper is available from the lead contact upon request.

### EXPERIMENTAL MODEL AND STUDY PARTICIPANT DETAILS

#### Yeast Strains, Plasmids and DNA Oligos

Yeast strains and plasmids used in this study are listed in key resources table. DNA oligos used are listed in Table S1. The HIS3 cassette was amplified from pFA6a-6xGLY-FLAG-HIS3.^95^ *CDC33-HIS3* and *cdc33-ts4-2-HIS3* (E73K, G179D) yeast strains were constructed in the BY4741 background (MATa; *his3Δ1*; leu2Δ0; met15Δ0; ura3Δ0) using standard PCR-based techniques. Plasmids pDB-CDC123, constructed by Gibson Assembly (New England Biolabs; cat# E2611S) using primers listed in Table S1, and pC2873 were transformed into BY4741 *CDC33-HIS3* and *cdc33-ts4-2-HIS3*. *CDC33-HIS3* and *cdc33-ts4-2-HIS3* strains were also constructed in the J292 background (*MAT*a *leu2-3,-112 ura3-52 his3 gcn2Δ::loxP gcd11Δ::KanMX* p[*GCD11, URA3*]).^54^ pC2872 was transformed into the *CDC33/cdc33-ts4-2* J292 background via plasmid shuffling to replace the [*GCD11*, *URA3*] plasmid.^110^ p713 and p722 were transformed into J292 *CDC33/cdc33-ts4-2* pC2872. pAG425-TIF1 was generated by Gibson Assembly using pAG425-GPD-ccdB (gift from Susan Lindquist; Addgene plasmid # 14154) as backbone and PCR based insert generated using primers listed in Table S1. pAG425-CAF20 was generated by Gateway Cloning. Plasmids were transformed by lithium acetate method^111^ or electroporation. Cells were either grown in YPD or synthetic complete medium with all amino acids except histidine/leucine or histidine and uracil. For cells treated with 3-Amino-1,2,4-triazole (3-AT; Millipore Sigma; cat# A8056), cells were grown at 25°C to OD ~0.5 in synthetic complete medium minus histidine/leucine or histidine and uracil, then treated with 100 mM 3-AT for an hour.

### METHOD DETAILS

#### Polysome Profiling

*CDC33* and *cdc33-ts4-2* cells were grown in YPD at 25°C to OD ~0.5. Cultures were split in two, with half remaining at 25°C and the other half shaken in a pre-warmed 37°C water bath. After an hour, cycloheximide was added to a final concentration of 100 μg/mL. After incubation with cycloheximide for 2 minutes, cells were pelleted, and flash frozen on dry ice. Cell pellets were resuspended in polysome-lysis buffer (20 mM Tris pH 7.5, 140 mM KCl, 1.5 mM MgCl_2_, 0.5 mM DTT, 100 μg/mL cycloheximide, 200 μg/mL heparin, 1% Triton), washed once, and lysed with glass beads using a FastPrep-24 (MP Biomedical). For RNAse treatment, supernatants from cleared lysates corresponding to ~20 A_260_ of total RNA were first treated with 300 U of RNase I (ThermoFisher Scientific; cat# AM2294) at 25°C for 1 hour. Lysates were then layered over 10–50% sucrose density gradients (SGD) and centrifuged for 2h 40 min (4°C) at 35,000 rpm on SW41Ti to separate ribosome protected RNA fragments. Pooled SGD fractions were further centrifuged for 2 h (4°C) at 267,000 × g over a sucrose cushion (1.1 M sucrose, 20 mM Tris pH 7.5, 500 mM NH_4_Cl, 10 mM MgCl_2_, and 0.5 mM EDTA pH 7.5) in an MLA-130 rotor (Eppendorf). RNA was extracted using a hot phenol method.^112^

#### mRNA PolyA Purification and Fragmentation

CNBr-activated Sepharose beads (Cytiva; cat# 17098101) were coupled to polydT_25_ using the method in Chockalingam et al.^113^ Total RNA was heated at 65°C, incubated with polydT beads in binding buffer (10 mM Tris pH 7.5, 400 mM NaCl, 1 mM EDTA, 0.1% SDS) at room temperature for 10 minutes, washed twice with wash buffer (10 mM Tris pH 7.5, 40 mM NaCl, 1 mM EDTA, 0.1% SDS), and eluted with 20 mM KOH. Eluted samples were neutralized via addition of 3 M sodium acetate pH 5.2 to a final concentration of 300 mM and ethanol precipitated. polyA selected RNAs were fragmented by incubation at 95°C for 20 minutes in fragmentation buffer (50 mM sodium bicarbonate pH 9.2, 1 mM EDTA). Reactions were stopped by the addition of 3 M sodium acetate pH 5.2 to a final concentration of 300 mM and samples were ethanol precipitated.

#### RNA-seq and Ribosome Profiling Library Preparation

Following PAGE purification and size selection (21–34 nt) on a 15% urea PAGE gel, ribosome-protected RNA fragments were subjected to ribosomal blanking by annealing with biotinylated primers (Table S1) and incubation with streptavidin beads (ThermoFisher Scientific; cat# 88816) following manufacturer’s instructions. Blanked ribosome protected fragments and fragmented mRNAs were dephosphorylated using T4 polynucleotide kinase (NEB; cat# M0201S). Fragments were then ligated to a short adenylated DNA oligonucleotide—5′rAppCTGTAGGCACCATCAAT/3ddC/3′—at their 3′ end using T4 RNA ligase 2, truncated (NEB; cat# M0242S). Ligated products were purified using denaturing urea PAGE and reverse transcribed using M-MuLV reverse transcriptase (NEB; cat# M0253L) and RS-1 primer (/5Phos/AGATCGGAAGAGCGTCGTGTAGGGAAAGAGT GTAGATCTCGGTGGTCGC/iSp18/CACTCA/iSp18/TTCAGACGTGTGCTCTTCCG ATCTATTGATGGTGCCTACAG. cDNA products were circularized using CircLigase (Lucigen; cat# CL4111K). Optimal amplification cycle number was determined via pilot PCR before PCR amplification with Phusion polymerase (NEB; cat# M0530S) and unique barcoded primers. DNA libraries were purified using native PAGE and then analyzed for length and purity using Agilent Bioanalyzer.

#### Sequencing and Quality Control of Reads

Prepared cDNAs were sequenced on a HiSeq 2500 at the Genome Technology Access Center (GTAC) of Washington University in St. Louis. Samples were demultiplexed based on their 6-nt barcode, allowing for 1 mismatch, using Flexbar 3.5^98^ and checked for initial quality using FastQC 0.11.9.^99^ Reads were then processed with Cutadapt 4.2^100^ to remove the 17-nt linker sequence, requiring at least 15 nt of overlap. For ribosome profiling reads, any reads not containing the linker were discarded, while for the RNA-seq reads, any reads containing the linker were discarded. rRNAs, tRNAs, snoRNAs, and other ncRNAs were filtered out by mapping to the R64-1-1 release ncRNA fasta file from SGD using Hisat2 2.2.1.^101^

#### Immunoblotting

Whole cell lysates were harvested and lysed in 1 mL of ice-cold lysis buffer (300 mM NaOH, 1% β-mercaptoethanol). Proteins were precipitated through the addition of TCA to 10% concentration by volume and resuspended in HU buffer (8 M Urea, 5% SDS, 200 mM Tris pH 6.8, 100 mM DTT, 1 mM ethylenediaminetetraacetic acid (EDTA), bromophenol blue) using a volume normalized to the harvested OD. Proteins were separated by SDS–PAGE and analyzed by immunoblotting. Antibodies used in this study are listed in key resources table. Working antibody dilutions for immunoblot analysis were made as per the manufacturer’s instructions.

#### Real-Time Quantitative Reverse Transcription PCR

Total RNA was isolated using a hot phenol method^112^ and treated with DNase I (ThermoFisher Scientific; cat# EN0521). M-MuLV reverse transcriptase (NEBcat# M0253L) was used to generate cDNA from ~2 ug of total RNA and random hexamers (ThermoFisher Scientific, cat# SO142) following manufacturer’s instructions. Quantitative RT-PCR was conducted using iTaq Universal SYBR Green Supermix (Bio-Rad; cat# 1725121) following manufacturer’s instructions.

#### Measurement of Renilla and Firefly Luminescence

Luminescence was measured as described in Simms et al.^114^ Briefly, cells were grown in synthetic complete medium minus uracil to OD ~0.5 at 25°C. Half the culture was then shifted to 37°C for an hour before both cultures were collected by centrifugation and washed once with TE. Cells were then resuspended in zymolyase buffer (50 mM Tris pH 7.5, 10 mM MgCl_2_, 1 M Sorbitol, 30 mM DTT) and incubated with lyticase from *Arthrobacter luteus* (Millipore Sigma; cat# L2524) at 37°C for 30 minutes. Cells were lysed by the addition of passive lysis buffer (Promega; cat# E1941). Samples were pelleted by centrifugation at 4000 × g for 5 minutes at room temperature and cleared lysates were transferred to 96-well plates. Luminescence was measured using the Dual-Luciferase Reporter Assay System (Promega; cat# E1910) following manufacturer’s instructions on an Infinite F200 Pro plate reader (Tecan).

#### Yeast β-Galactosidase Activity

Yeast strains harboring the GCN4-*lacZ* reporters were grown in synthetic complete medium (-Ura, -His), supplemented with adenine, to mid-log phase (OD_600_ 0.6–0.7). Cultured cells were then split into 2 groups: untreated and 3-AT treated (100 mM 3-aminotriazole for 1h). For heat shock treatments, 5 mL of cells were grown in YAPD at 25°C to their mid log phase, and half the cultures were shifted to 37°C for an hour. Following lysis, as described earlier, 50 μL of the cleared lysate was aliquoted into a new tube, and 100 μL of Buffer Z (0.06M Na_2_HPO_4_, 0.04 M NaH_2_PO_4_, 0.01 M 1M KCl, 0.001M 1M MgSO_4_) containing β-mercaptoethanol (3.5 μL/ml of buffer Z), and 30 μl of 4 mg/ml o-nitrophenyl-β-D-galactopyranoside (ONPG) in Buffer Z was added. Reaction mixture was incubated at 28°C for 30–60 min. Reactions were then stopped by adding equal volume of 1M Na_2_CO_3_, transferred to a clear 96-well plate and absorbance at 420 nm and 550 nm was measured using an Infinite M200 Pro plate reader (Tecan).

#### Puromycin incorporation and Immunoblot Analysis

To assess overall protein synthesis, *in vivo* incorporation of puromycin into nascent peptides was evaluated by immunoblot analysis. Briefly, cells were grown to mid log phase (0.6–0.7) and treated with 1 mM puromycin directly adding to the culture media for 30 minutes. Cells were then rapidly pelleted by centrifugation (5000 × g for 2 min). Samples were processed for immunoblot as described earlier.

#### Northern Blot Analysis

*CDC33* and *cdc33-ts4-2* cells were grown to OD_600_ 0.6–0.7 at 26°C. Similar to immunoblot analysis, cells were then split into 2 groups – half the cultures were continued to grow at 26°C, while the other half was shifted to 37°C for an hour. Cells from both groups were immediately pelleted by centrifugation, washed with AE buffer (50 mM NaOAC pH 5.2, 10 mM EDTA) and flash frozen on dry ice. RNA was isolated from frozen cell pellets by hot acid phenol/chloroform extraction method as described earlier.^66,112^ 5 μg of total RNA was resolved on 1% formaldehyde agarose gel to check the quality of RNA. For northern analysis, 5μg of total RNA was separated on 7 M Urea 8% acrylamide (19:1). Nucleic acids were transferred onto positively charged Zeta-Probe Blotting membranes (BioRad) using a Trans-Blot semi-dry transfer apparatus (BioRad). Following transfer, nucleic acids were UV cross-linked to the membrane for 10 min (Energy: 100μ J/cm^2^) and baked at 80°C for 15 minutes. After crosslinking, the membrane was placed in a glass hybridization bottle, 15 mL of Sigma PerfectHyb Plus Hybridization Buffer (cat# H7033) was added and incubated in hybridization oven for 60 min at 42°C to block the membrane. A radiolabeled DNA probe, labelled using polynucleotide kinase and [γ-32P]-ATP (Perkin Elmer), was then added and incubated overnight. Membranes were washed with non-stringent buffer (2 × SSC, 0.1% SDS) three times, followed by three washes in stringent buffer (0.2 × SSC, 0.1% SDS), all at hybridization temperatures for 15 min. Membranes were then exposed to a phosphor imager screen and analyzed using Amersham Typhoon laser scanner (Cytiva).

### QUANTIFICATION AND STATISTICAL ANALYSIS

#### Quantification of Immunoblots and Northern Blots

Intensities of bands were quantified using ImageQuant TL (Cytiva). For immunoblots, band intensities were normalized to the intensity of the corresponding Pgk1 band (Figures 1B, 1D, 6B, 7B, 7E, 7H, 7L, S5, and S6). For northern blots, band intensities were normalized to the intensity of the corresponding 5S rRNA band (Figure S4B). Plotting of graphs and statistical analysis was done in GraphPad Prism 8.4.3 (GraphPad Software, La Jolla, CA). Plots display the mean of at least three independent experiments, with error bars representing the standard deviation around the mean. p-values were calculated using the two-tailed, unpaired t-test function in Prism, with the significance threshold set at p ≤ 0.05.

#### Quantification of Real-Time Quantitative Reverse Transcription PCR

The fold change for each gene was calculated by using the ΔΔCt method; expression at 37°C was normalized to expression of *TAF10*, then compared to the corresponding value calculated at 25°C (Figure 5C). Plotting of graphs and statistical analysis was done in Prism. Plots display the mean of at least three independent experiments, with error bars representing the standard deviation around the mean. p-values were calculated using the two-tailed, unpaired t-test function in Prism, with the significance threshold set at p ≤ 0.05.

#### Quantification of Luminescence

Firefly luciferase luminescence was normalized to renilla luciferase luminescence (Figure S2D). Plotting of graphs and statistical analysis was done in Prism. Plots display the mean of at least three independent experiments, with error bars representing the standard deviation around the mean.

#### Quantification of β-Galactosidase Activity

Enzyme activity was calculated by Miller Units = 1000 × [(Abs_420_ – (1.75 × Abs_550_)] / (T 3 V × OD_600_), which presents change in Abs_420_/min/mL of cells/OD_600_ (Figures 6D, 7C, 7F, 7I, 7J, and S2B). Plotting of graphs and statistical analysis was done in Prism. Plots display the mean of at least three independent experiments, with error bars representing the standard deviation around the mean. p-values were calculated by the two-tailed, unpaired t-test function in Prism, with the significance threshold set at p ≤ 0.05.

#### Analysis of Genome Mapped Reads

Filtered reads were mapped to the R64-1-1 genome (SGD) using STAR 2.7.10b,^102^ allowing for 2 mismatches in the RNA-seq reads or 1 mismatch in the ribosome profiling reads, with only uniquely mapping reads kept. Output sam files were converted into bam files using Samtools 1.16.1.^103,104^ Ribosome profiling reads were uploaded to the RiboA webtool^115^ and analyzed for frame using the “sacCer3_R64-2-1_20150113.gff” annotation, “sacCer3_R64-2-1_genome.fa” fasta file, and quantification from the 3′ end, with all other options kept as their default (Figure 2A). Bam files were analyzed using FeatureCounts 2.0.1^105^ to count reads mapping to 5′ UTRs, CDSes, introns, and 3′ UTRs with strandedness enforced and requiring at least 50% of the read to map to the feature (Figure 2B). Features were annotated in a custom R64-1-1 (SGD) annotation file with the addition of 5′ and 3′ UTR annotations from the Pelechano study,^116^ using the longest UTR, or default 5′ and 3′ UTRs of 120 and 200 nt, respectively. Reads mapping to 5′ UTRs, introns, and 3′ UTRs were normalized by feature length, and then normalized again by the average coverage of the corresponding CDS. All analyzed features were filtered for outliers using the ROUT method in GraphPad Prism with Q = 0.1%. Bam files were also converted to bed files and coverage across unique, non-overlapping features was counted using Bedtools 2.30.0,^106^ with reads containing introns mapped as independent fragments and matching strandedness enforced (Figures 2C and 5A). For ribosome profiling reads, coverage was determined using a “pseudo-A” site coordinate, which was calculated by taking the midpoint of the mapped read coordinates, with weighting towards the 5′ end of the fragment if the midpoint fell between bases (Figures 2C and 5A). Coverage was then extracted for all genes with 5′ UTR, CDS, and 3′ UTR of at least 100 nt in length and at least 128 reads mapping to the CDS (Figure 2C). The coverage at each position was normalized by the mean coverage across the gene (Figure 2C). Coverage corresponding to each feature was divided evenly across 100 bins and averaged among all features of the same type across all analyzed genes (Figure 2C). Coverage calculations were done in custom Python scripts using Biopython^117^ and SciPy (Figure 2C).^118^

#### Analysis of Transcriptome Mapped Reads

Filtered reads were mapped to the transcriptome using Salmon 1.9.0,^39^ with 50 nt upstream and downstream of annotated CDSes included and the whole genome used as a decoy. Reads were mapped with the stranded forward (SF) library option, k-mer values of 11 and 21 for the ribosome profiling and RNA-seq reads, respectively, and fldMean and fldSD values taken from the Bioanalyzer results for each sample (Table S2). Salmon quantified reads were converted into a count matrix and imported into DESeq2 1.32.0^40^ using tximport 1.20.0.^107^ Counts were transformed using a variance-stabilizing transformation method with blind set to true. Transformed counts were plotted on a heatmap with clustering by Euclidean distances (Figure 3A), as well as subjected to principal component analysis (Figure 3B), using the base functions in R 4.2.1.^108^ Differential gene expression was determined for all combinations of strain and temperature with DESeq2 using the Wald test with Benjamini-Hochberg correction. All reported Log_2_ fold changes were first shrunk using the ashr algorithm.^119^ The comparisons tested in DESeq2 can be found in the Zenodo repository. Differentially expressed genes with adjusted p-value ≤0.05 and annotated as upregulated by Hsf1, Msn2, and Msn4 on Yeastract+^120^ using the settings “TF acting as activator” and “DNA binding and expression evidence” were plotted by their Log_2_ fold change in Prism (Figure 3C). All genes output from the DESeq2 analysis were plotted in volcano plots in Prism (Figure 4A).

#### Downstream Bioinformatic Analysis

For analysis of Kozak sequence context (Figure 4B), the first six nucleotides upstream and downstream of the start codon were extracted for all coding genes using the “orf_coding” and “orf_genomic_1000” sequences for the R64-1-1 annotation from SGD. Sequences from coding genes corresponding to either the 100 most upregulated TEs, or the 100 most downregulated TEs, with adjusted p-value ≤0.05, were analyzed against the sequences from all genes using STREME,^121^ with default settings except: “Convert DNA to RNA”, Minimum Motif Width 9, and Maximum Motif Width 15. For the Pearson correlation matrix (Figure 4C), GC content was calculated using the sequence of the whole transcript (CDS plus 5′ UTR and 3′ UTR sequences from the Pelechano annotation, or default 5′ and 3′ UTR sequences of length 120 and 200, respectively, extracted from the genome). ΔG was calculated using the same sequences from above using the ViennaRNA Package 2.5.1.^109^ Genes marked as part of the Gcn4 regulon were classified based on the UC and T dataset in Rawal et al.^122^ For gene ontology analysis (Figure 4D), the sequences of all transcripts (CDS plus 5′ UTR and 3′ UTR sequences from the Pelechano annotation, or default 5′ and 3′ UTR sequences of length 120 and 200, respectively, extracted from the genome) showing a Log_2_ fold change >= 1 in response to loss of eIF4E were compiled into a fasta file and analyzed using STREME^121^ from the online MEME suite with default settings. The top 100 upregulated genes as a result of loss of eIF4E in the RNA-seq and Ribosome Profiling datasets were analyzed using the Gene Ontology Term Finder on SGD (v 0.86) with default settings.

## Supplementary Material

MMC3

MMC2

MMC1

## Figures and Tables

**Table:** KEY RESOURCES TABLE

REAGENT or RESOURCE	SOURCE	IDENTIFIER
Antibodies		
Monoclonal ANTI-FLAG M2 peroxidase (HRP) Antibody produced in mouse	Sigma Aldrich	cat# A8592; RRID:AB_439702
HA-Tag (6E2) Mouse mAb (HRP Conjugate)	Cell Signaling Technology	cat# 2999S; RRID:AB_1264166
His-probe (H-3)	Santa Cruz Biotechnology	cat# sc-8036; RRID:AB_627727
Anti-Puromycin Antibody, clone 12D10 from mouse	Sigma-Aldrich	cat# MABE343; RRID:AB_2566826
Phospho-eIF2α (Ser51) (D9G8) Rabbit mAb	Cell Signaling Technology	cat# 3398S; RRID:AB_2771064
PGK1 Monoclonal Antibody (22C5D8)	Invitrogen	cat# 459250; RRID:AB_2532235
HRP Anti-PGK1 Antibody (22C5D8)	Abcam	cat# ab197960; RRID:AB_2756444
Goat anti-Rabbit IgG (H+L) Secondary Antibody, HRP	ThermoFisher Scientific	cat# 31460; RRID:AB_2533967
Goat anti-Mouse IgG (H+L) Secondary Antibody, HRP	ThermoFisher Scientific	cat# 31430; RRID:AB_228307
Rabbit anti-eIF4A1	Abcam	cat# ab31217
anti-Gcn4 Rabbit mAb	Hinnebusch Lab	N/A
Chemicals, peptides, and recombinant proteins		
Phusion High Fidelity DNA polymerase	NEB	cat# M0530S
Phusion Hot Start II DNA Polymerase	ThermoFisher Scientific	cat# F549L
iTaq Universal SYBR Green Supermix	BIO-RAD	cat# 1725121
M-MuLV reverse transcriptase	NEB	cat# M0253L
Random hexamers	ThermoFisher Scientific	cat# SO142
2X Gibson Assembly Master Mix	NEB	cat# E2611S
DNase I	ThermoFisher Scientific	cat# EN0521
RNAse I	ThermoFisher Scientific	cat# AM2294
T4 polynucleotide kinase	NEB	cat# M0201S
CircLigase	Lucigen	cat# CL4111K
T4 RNA ligase 2, truncated	NEB	cat# M0242S
Lyticase from *Arthrobacter luteus*	Millipore Sigma	cat# L2524
Passive lysis buffer	Promega	cat# E1941
Dual-Luciferase Reporter Assay System	Promega	cat# E1910
SuperSignal West Femto Maximum Sensitivity Substrate	ThermoFisher Scientific	cat# 34096
SuperSignal West Pico PLUS Chemiluminescent Substrate	ThermoFisher Scientific	cat# 34580
CNBr-Activated Sepharose	Cytiva	cat# 17098101
Streptavidin beads	ThermoFisher Scientific	cat# 88816
Cycloheximide ultra-pure	VWR	cat# 94271
Methyl methane sulfonate	Sigma Aldrich	cat# 129925
3-Amino-1,2,4-triazol	Sigma Aldrich	cat# A8056
2-Nitrophenyl β-D-galactopyranoside	Sigma Aldrich	cat# N1127
PerfectHyb Plus Hybridization Buffer	Sigma Aldrich	cat# H7033
Deposited data		
Raw and analyzed RNA-seq and ribosome profiling data	This paper	GSE223465
Custom python script to obtain values for Figure 1E	This paper	Zenodo: https://doi.org/10.5281/zenodo.7617427
Raw gel image files	This paper	Mendeley Data: https://doi.org/10.17632/gg5zwy34bc.1
Experimental models: Organisms/strains		
BY4741 (*MATa his3Δ1 leu2Δ0 met15Δ0 ura3Δ0*)	Dharmacon lab	N/A
BY4741 *CDC33* (*BY4741; CDC33-HIS3*)	This Study	N/A
BY4741 *cdc33-ts4-2* (*BY4741; cdc33-ts4-2-HIS3*)	This Study	N/A
J292 (*MATα leu2-3, -112 ura3-52 his3 gcn2Δ::loxP gcd11Δ::KanMX GCD11-URA3*)	Alone et al.^54^	N/A
J292 *CDC33-HIS3* (*J292; CDC33-HIS3*)	This Study	N/A
J292 *cdc33-ts4-2* (*J292; cdc33-ts4-2-HIS3*)	This Study	N/A
1091 (BY4741; *GCN4-3XFLAG-KAN; CDC33-HA-HIS3*)	This Study	N/A
1093 (BY4741; *GCN4-3XFLAG-KAN; cdc33-ts4-2-HA-HIS3*)	This Study	N/A
1143 (1091; *tif1Δ::LEU2*)	This Study	N/A
1148 (1093; *tif1Δ::LEU2*)	This Study	N/A
YAS1955 (MATa; *ade2-1; his3-11,15; leu2-3; ura3-1 trp1-1 pep4::HIS3; tif4631::LEU2; tif4632::ura3; pHA-TIF4632 TRP1*)	Watanabe et al.^64^	N/A
KAY109 (YAS1955; p*TIF4632-430*; L428A, L429A)	Watanabe et al.^64^	N/A
1324 (YAS1955; *GCN4-XFLAG-KAN*)	This study	N/A
1326 (KAY109; *GCN4-XFLAG-KAN*)	This study	N/A
Oligonucleotides		
DNA oligos used for cloning: See Table S1	N/A	N/A
Recombinant DNA		
pFA-CDC33-HIS3 (pFA6a-6xGLY-FLAG-HIS3MX6)	Funakoshi and Hochstrasser^95^	N/A
pDB-RL-X-FL (Renilla-Firefly luciferase fusion construct with an in-frame stop codon placed between; constructed with the pDB688 backbone)	This Study; pDB688 Salas-Marco and Bedwell^96^	N/A
pDB-RL-X-GCN4-FL (Same as pDB-RL-X-FL except the first 60 nt of the *GCN4* coding sequence placed after the in-frame stop codon; constructed with the pDB688 backbone)	This Study; pDB688 Salas-Marco and Bedwell^96^	N/A
pDB-RL-X-5′ UTR-GCN4-FL (Same as pDB-RL-X-GCN4-FL except the 5′ UTR of *GCN4* with all four uORFs placed between the in-frame stop codon and the first 60 nt of the *GCN4* coding sequence; constructed with the pDB688 backbone)	This Study; pDB688 Salas-Marco and Bedwell^96^	N/A
pDB-RL-GCN4-FL (Renilla-Firefly luciferase fusion construct with the first	This Study; pDB688 Salas-Marco and Bedwell^96^	N/A
60 nt of the *GCN4* coding sequence placed between; constructed with the pDB688 backbone)		
pDB-CDC123 (_GPD_ CDC123-HA-URA3; constructed with the pDB688 backbone)	This Study; pDB688 Salas-Marco and Bedwell^96^	N/A
pC2872 (*His*_*8*_*-GCD11 (eIF2*γ*), LEU2, CEN4/ARS* )	Alone et al.^54^	N/A
pC2873 (*His*_*8*_*-GCD11 (eIF2*γ*), LEU2, pRS425*)	Alone et al.^54^	N/A
P713 (*URA3, CEN6*) Wek et al.^97^	N/A	
p722 (*GCN2, URA3, CEN6*)	Wek et al.^97^	N/A
p180 (*lacZ-GCN4, URA3*)	Hinnebusch^57^	N/A
p226 (p180, uORFs 1, 2 and 3 removed)	Abastado et al.^56^	N/A
p227 (p180, all uORFs removed)	Abastado et al.^56^	N/A
p466 (p180, uORFs 2, 3 and 4 removed)	Abastado et al.^56^	N/A
pA44 (p180, uORFs 2 and 3 removed)	Abastado et al.^56^	N/A
pA46 (pA44, stem loop inserted between uORF 1 and 4)	Abastado et al.^56^	N/A
pA50 (pA44, stem loop inserted downstream of uORF 4)	Abastado et al.^56^	N/A
pA61Z (146 nt inserted between uORF1 and uORF4)	Abastado et al.^56^	N/A
pAG-TIF1 (TIF1 genomic region including promoter and UTR cloned into pAG425GPD-ccdB, by removing GPD promoter and CYC1 3′ UTR.)	This study; Addgene plasmid # 14154	N/A
pAG-CAF20 (CAF20 (S91A and T102A) inserted into pAG425GPD-ccdB using the gateway recombination approach)	This study; Addgene plasmid # 14154	N/A
Software and Algorithms		
HiSeq	Illumina	Version 2500
Flexbar	Roehr et al.^98^	Version 3.5
FastQC	Andrew^99^	Version 0.11.9
Cutadapt	Martin^100^	Version 4.2
Hisat2	Kim et al.^101^	Version 2 2.2.1
STAR	Dobin et al.^102^	Version 2.7.10b
Samtools	Danecek et al.^103^; Li et al.^104^	Version 1.16.1
FeatureCounts	Liao et al.^105^	Version 2.0.1
Bedtools	Quinlan and Hall^106^	Version 2.30.0
Salmon	Patro et al.^39^	Version 1.9.0
DESeq2	Love et al.^40^	Version 1.32.0
tximport	Soneson et al.^107^	Version 1.20.0
R	R Core Team^108^	Version 4.2.1
ViennaRNA Package	Lorenz et al.^109^	Version 2.5.1
ImageQuant TL	Cytiva	Version 7.0
GraphPad Prism	GraphPad	Version 8.4.3

## Highlights

- Depletion of eIF4E leads to translation of *GCN4* and induction of its targets
- *GCN4* translation requires ribosome scanning independently of eIF2α phosphorylation
- Ternary complex concentration does not change in the absence of eIF4E
- The relative concentration of eIF4A to eIF4E and/or eIF4G regulates *GCN4* translation
