# Supplementary material for: eIF4F complex dynamics are important for the activation of the integrated stress response
Source: Mol Cell. Author manuscript; Available in PMC 2024 Jun 20. (PMC11189614; doi:10.1016/j.molcel.2024.04.016)

Molecular Cell, Volume *84*

Supplemental information

eIF4F complex dynamics are important for the activation of the integrated stress response

Kyusik Q. Kim, Ankanahalli N. Nanjaraj Urs, Victor Lasehinde, Alison C. Greenlaw, Benjamin H. Hudson, and Hani S. Zaher

Supplemental information

The stoichiometry of the eIF4F complex is important for the activation of the integrated stress response

Kyusik Q Kim, Ankanahalli N Nanjaraj Urs, Victor Lasehinde, Alison C Greenlaw, Benjamin H Hudson, and Hani S Zaher

Supplementary Figures Legends

Figure S1: Cycloheximide addition does not appear to significantly alter ribosome occupancy per gene measurements during ribosome profiling, related to Figure 3.

Analysis of the data from[1]. Data are stored in the Gene Expression Omnibus under accession number GSE59573. RPKM values from the GSE59573_Expression_Table.txt file was plotted and analyzed using Graphpad Prism. For the Unstressed comparison, unstressed_noCHX (Sample_1) was plotted against unstressed_1x_CHX (Sample_5), and the Pearson correlation coefficient (r) calculated using the correlation function with default settings. For the heatshock comparison, the same was done except plotting heatshock_noCHX (Sample_17) against heatshock_1x_CHX (Sample_18).

Figure S2: Ribosome scanning is important for *GCN4* translation de-repression when eIF4E is depleted, related to Figure 6.

A) Schematics of the *GCN4-lacZ* reporters used to study *GCN4* translation de-repression. **B)** Bar graphs summarizing *lacZ* expression in *CDC33* and *cdc33-ts4-2* cells from the depicted reporters under the indicated conditions. **C)** Diagram of the dual-luciferase reporter construct. **D)** Firefly luciferase luminescence normalized to renilla luciferase luminescence for the indicated reporter constructs and conditions. Cells were grown in synthetic complete medium minus uracil to $OD_{600\ nm}$ ~0.5 at 25°C. Half the culture was shifted to 37°C for an hour before both cultures were collected. For reporter constructs: X refers to an in-frame stop codon placed between luciferases, X-GCN4-fus refers to an in-frame stop codon plus the first 60 nt of the *GCN4* coding sequence, while X-5'UTR-GCN4 refers to an in-frame stop codon plus the 5'UTR of *GCN4* with all four uORFs included, along with the first 60 nt of the *GCN4* coding sequence. A reporter construct with only the first 60 nt of the *GCN4* coding sequence placed between luciferases (GCN4-FLfus) was used as a positive control. Plotted are the average values of three biological replicates with error bars representing the standard deviation around the mean.

Figure S3: Overexpression of eIF2γ or CDC123 does not lead to accumulation of Gcn4, related to Figure 6.

A) and **B)** Immunoblot analysis used to assess *GCN4* induction in the indicated cells and under the depicted conditions, when *GCD11* and *CDC123* are overexpressed, respectively.

Figure S4: tRNA levels do not change when eIF4E is depleted, related to Figure 6.

A) Representative phosphorimager scans of northern blots used to assess the levels of the shown RNA species isolated from the indicated cells grown under the indicated conditions. The bottom shows UV scan of the gels, prior to transfer, stained with ethidium bromide. **B)** Bar graphs summarizing the quantification of the phosphorimages used to determine the relative levels of the indicated tRNA species to 5S rRNA in the depicted strain grown under the indicated conditions. Plotted is the average of four independent experiments with the error bars representing the standard deviation around the mean.

Figure S5: Global protein synthesis is not imapcted upon Tif1 (eIF4A) depletion, related to Figure 7.

Immunoblot analysis of puromycin incorporation in WT and its *tifΔ* cells. The quantification of blots from three biological repeats is plotted on the right. The error bars represent the standard deviation around the mean. p values determined by unpaired parametric t test, are plotted above the values being compared.

Figure S6: eIF2α phosphorylation does not change when eIF4A is overexpressed, related to Figure 7.

Bar graph summarizing the quantification of three independent immunoblots, similar to the one shown in Figure 7G, used to assess the levels of P-eIF2α in wild type cells harboring an empty vector or a *TIF1*-overexpressing plasmid in the absence and presence of 3-AT. Plotted is the average of the three independent experiments with the error bars representing the standard deviation around the mean.

Figure S7: Overexpression of Caf20 results in accumulation of Gcn4 in *cdc33-ts4-2* cells under permissive temperature, related to Figure 7.

Immuno blot analysis used to assess the levels of GCN4 in CDC33 and the *cdc33-ts4-2* cells having either empty plasmid control or CAF20 (encoding eIF4E-BP) at 25 ºC and 37ºC.

References

1. Gerashchenko, M.V., and Gladyshev, V.N. (2014). Translation inhibitors cause abnormalities in ribosome profiling experiments. Nucleic Acids Res *42*, e134. 10.1093/nar/gku671.

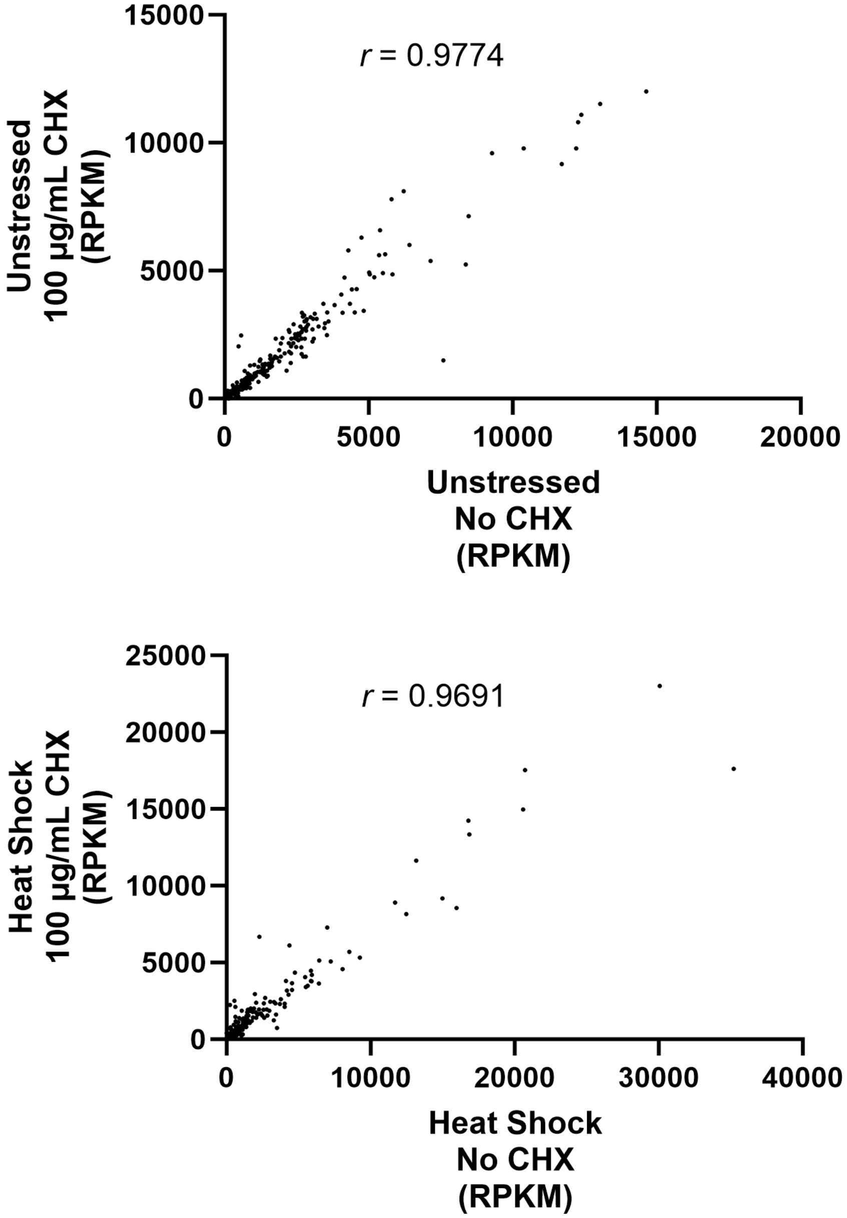
r = 0.9774
Unstressed
100 µg/mL CHX
(RPKM)
15000
10000
5000
0
0
5000
10000
15000
20000
Unstressed
No CHX
(RPKM)
r = 0.9691
Heat Shock
100 µg/mL CHX
(RPKM)
25000
20000
15000
10000
5000
0
0
10000
20000
30000
40000
Heat Shock
No CHX
(RPKM)

A

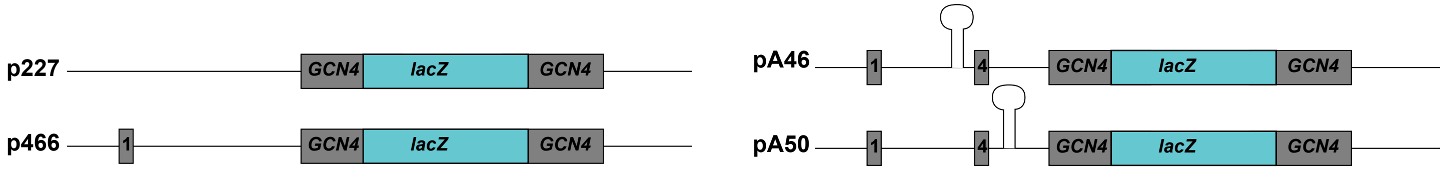
p227
GCN4
lacZ
GCN4
p466
1
GCN4
lacZ
GCN4
pA46
1
4
GCN4
lacZ
GCN4
pA50
1
4
GCN4
lacZ
GCN4

B

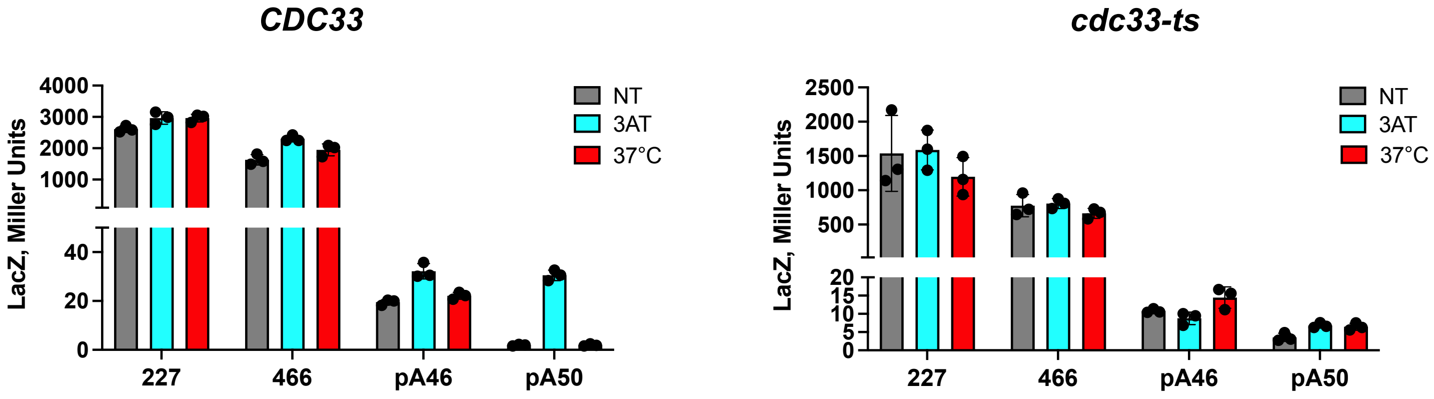
CDC33
LacZ, Miller Units
4000
3000
2000
1000
40
20
0
NT
3AT
37°C
227
466
pA46
pA50
cdc33-ts
LacZ, Miller Units
2500
2000
1500
1000
500
20
15
10
5
0
NT
3AT
37°C
227
466
pA46
pA50

C

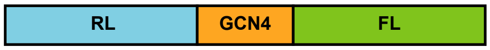
RL
GCN4
FL

D

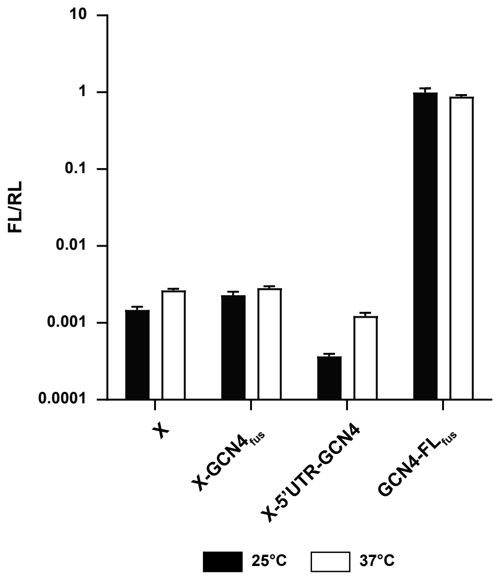
FL/RL
10
1
0.1
0.01
0.001
0.0001
X
X-GCN4fus
X-5'UTR-GCN4
GCN4-FLfus
25°C
37°C

Kim et al. Figure S3

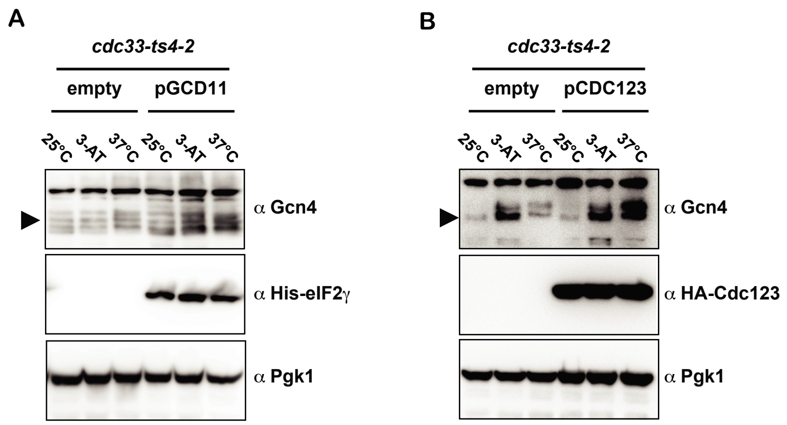

Kim et al. Figure S4

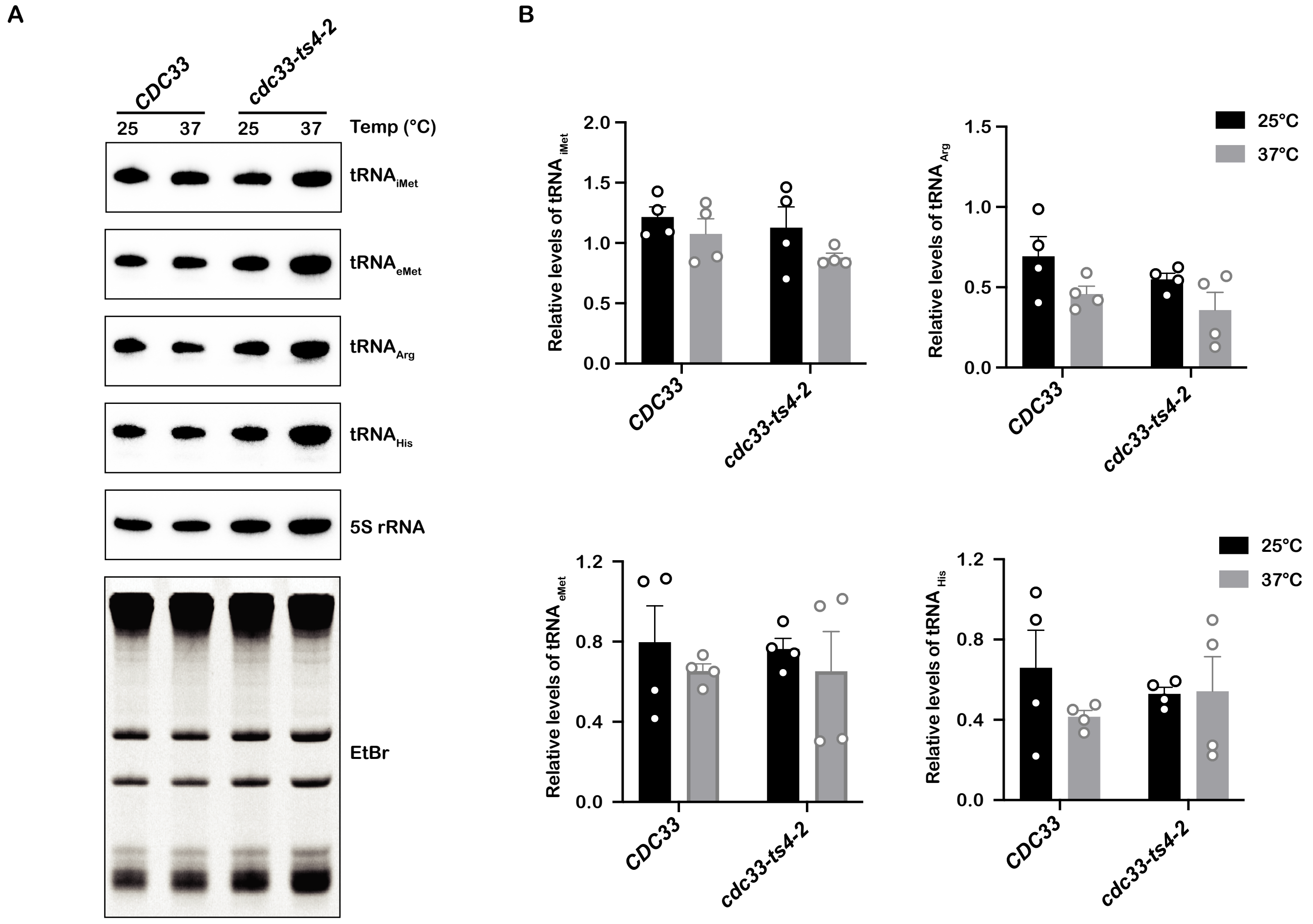

Kim et al. Figure S5

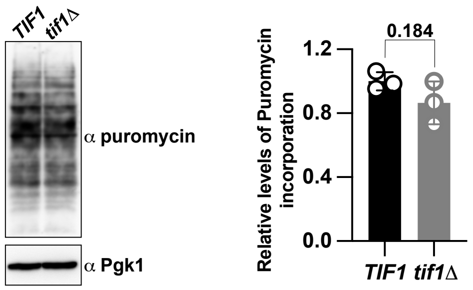

Kim et al. Figure S6

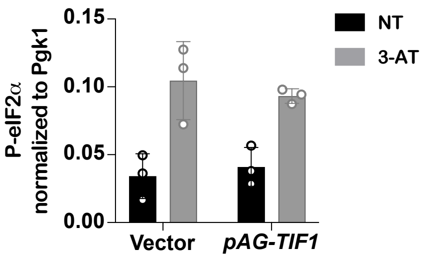

Kim et al. Figure S7

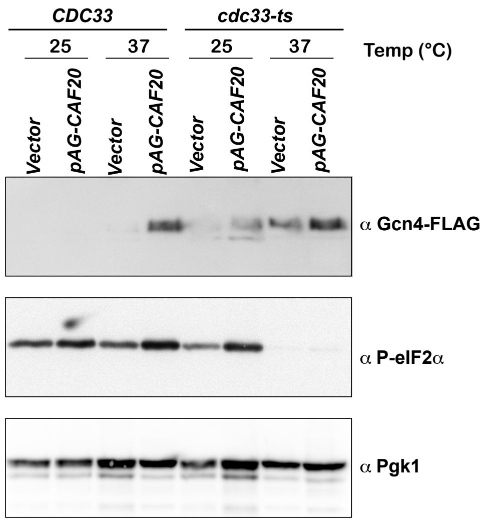

Supplement: MMC1 [file NIHMS1990599-supplement-MMC1.pdf]
